# Effects of malaria volunteer training on coverage and timeliness of diagnosis: a cluster randomized controlled trial in Myanmar

**DOI:** 10.1186/1475-2875-11-309

**Published:** 2012-09-04

**Authors:** Ohnmar, Tun-Min, San-Shwe, Than-Win, Virasakdi Chongsuvivatwong

**Affiliations:** 1 Department of Medical Research (Lower Myanmar), Yangon, Myanmar; 2 Department of Health, Vector Borne Disease Control Programme, Bago Division, Myanmar; 3 Department of Health, Nay Pyi Taw, Myanmar; 4 Epidemiology Unit, Faculty of Medicine, Prince of Songkla University, Hat Yai, Thailand

**Keywords:** Fever, Malaria, Early diagnosis, Artemisinin-based combination therapy, Volunteer workers, Mortality, Myanmar

## Abstract

**Background:**

The use of community volunteers is expected to improve access to accurate diagnosis and timely treatment of malaria, using rapid diagnostic test (RDT) and artemisinin-based combination therapy (ACT). However, empirical data from the field are still limited. The aim of this study was to assess whether training village volunteers on the use of Paracheck-Pf*®* RDT and ACT (artemether-lumefantrine (AL)) for *Plasmodium falciparum* and presumptive treatment with chloroquine for *Plasmodium vivax* had an effect on the coverage of timely diagnosis and treatment and on mortality in malaria-endemic villages without health staff in Myanmar.

**Methods:**

The study was designed as a cluster randomized controlled trial with a cross-sectional survey at baseline, a monthly visit for six months following the intervention (village volunteers trained and equipped with Paracheck-Pf*®*) and an endline survey at six months follow-up. Survey data were supplemented by the analysis of logbooks and field-based verbal autopsies. Villages with midwives (MW) in post were used as a third comparison group in the endline survey. Intention-to-treat analysis was used.

**Results:**

Of 38 villages selected, 21 were randomly assigned to the intervention (two villages failed to participate) and 17 to the comparison group. The two groups had comparable baseline statistics. The blood tests provided by volunteers every month declined over time from 279 tests to 41 but not in MW group in 18 villages (from 326 to 180). In the endline survey, among interviewed subjects (268 intervention, 287 in comparison, 313 in MW), the coverage of RDT was low in all groups (14.9%, SE 2.4% in intervention; 5.7%, SE 1.7% in comparison; 21.4%, SE 2.6% in MW) although the intervention (OR 3.2, 95% CI 1.5-6.7) and MW (OR 5.4, 95% CI 2.6-11.0) were more likely to receive a blood test. Mean (SE) of blood tests after onset of fever in days was delayed (intervention 3.6 (0.3); comparison 4.8 (1.3); MW 3.2 (0.4)). Malaria mortality rates per 100,000 populations in a year were not significantly different (intervention 130 SE 37; comparison 119 SE 34; MW 50 SE 18). None of the dead cases had consulted volunteers.

**Conclusions:**

The results show that implementing volunteer programmes to improve the coverage of accurate and timely diagnosis with RDT and early treatment may be beneficial but the timeliness of detection and sustainability must be improved.

## Background

Late diagnosis and inappropriate treatment of malaria results in complications and mortality [1,2]. For uncomplicated *Plasmodium falciparum* malaria, the World Health Organization (WHO) recommends early diagnosis by microscopy or with a rapid diagnostic test (RDT) prior to treatment [3,4] with artemisinin-based combination therapy (ACT) [5]. Using RDT reduces the overuse of anti-malarial drugs [6-8]. The six-dose artemether-lumefantrine (AL) regimen recommended for adults and children with a body weight above or equal to 5 kg rates high on efficacy, safety, community acceptability, and treatment compliance. Studies of ACT have shown a significant reduction in the burden of malaria in Africa [4,5,9-12].

Conclusive information on the use of trained community volunteers to expand ACT remains limited [13]. Following small-scale training on the use of RDT in the Philippines [14], Myanmar [15] and Cambodia [16], the training of community volunteers has been advocated [17-25]. Studies in Sudan, Uganda and Nigeria [20,22,24] have advocated recruiting volunteers with high community acceptability. A three-day training of village volunteers on common disease management in the Lao People's Democratic Republic increased the performance of RDT and use of ACT [21]. RDT by village volunteers, integrated with other control activities, reduced malaria morbidity and mortality in Thailand and Ethiopia [19,23]. On the other hand, a cluster randomized trial in children under five years of age in Zambia showed a reduction in the overuse of anti-malarials, albeit with higher mortality in the intervention group [25]. Adverse consequences have also been reported in Nigeria, with 7.6% emergency readmission of patients and 13 confirmed deaths among 1,028 patients treated by volunteers [24]. Poor compliance with treatment by patients and insufficient referral to health staff were reported in Sierra Leone [26] while low compliance in the use of ACT for RDT-positive patients was reported in Congo [17]. A study in Zambia found high attrition, low performance and inadequate medical supplies [27] while Uganda reported a high retention of health volunteers with probable decline in child mortality [28]. Available evidence highlights the need for a careful review of volunteer programmes.

Myanmar is among 31 countries with the highest burden of malaria [29]. Over 25 million people were living in high-risk areas with an approximate ACT coverage of 43% but there has been limited evidence of a reduction in cases between 2000 and 2009 [4,29]. The World Malaria Report (2010) under preparation, cites 121,636 malaria cases due to *P. falciparum* and 40,167 to *Plasmodium vivax,* respectively and reports 972 malaria deaths [4]. Since 2006, the National Vector Borne Disease Control Programme (VBDC) has provided training and supplied RDT and ACT to village midwives in all 324 Myanmar townships. In rural areas however, a midwife often covers six to 11 villages and the availability of health personnel is a serious bottleneck for the programme. Since 2004, the Myanmar Council of Churches has implemented a community-based malaria control project focusing on early diagnosis and treatment using volunteers in 160 remote villages in eight townships [30]. While one study reports positive impact, a thorough programme evaluation has not been conducted.

The primary objective of this study was to determine whether the training of village volunteers and their regular supervision by health staff improve the coverage of timely diagnosis and treatment of malaria, to lower malaria-related mortality.

Secondary objectives were (i) to compare the prevalence of malaria-induced fever in three groups of villages; (ii) to assess villagers’ knowledge of malaria transmission and of the free blood tests available; (iii) to compare trends in patients tested by volunteers and midwives; and, (iv) to determine RDT-positive rates for different age groups in selected areas.

## Methods

### Study area, villages involved in the trial and participants

The study was conducted in Bago, a division in the south of Myanmar with 12,941 sq km of forested land and a population of 5,313,613 inhabitants. With an annual rainfall of 3,291 mm and temperatures ranging from 20.9°C and 32.3°C, malaria is endemic all year round with a peak in transmission in June and July. Six townships with moderate endemicity (1–10 per 1,000 population) were selected for this study (Bago, Daik-U, KyaukTaGar, Oktwin, Taungoo and YeDarShay). In 2008, 21 malaria deaths were reported by the six township hospitals but the number of deaths in patients who did not arrive at the hospitals is unknown. No volunteers had previously been trained in Bago Division.

Farming and forest work are the most common occupations of villagers in the study area although seasonal work in plantations (teak, sugar cane and rubber) or construction sites is frequent (water dams, road construction sites and mines).

Sketch maps were available in some health centres but villages were not indicated on the maps, and Global Position System (GPS) points were not available. In successive consultations with approximately 200 midwives, information for the study was collected including village maps, distance between villages and information about villages under their care. Villages with fewer than 70 households and those where midwives were stationed were excluded from the trial, as were villages presenting serious security problems. Only those remaining villages classified as “Stratum 1a” were included in the study (highest level of endemicity as per malaria control programme classification to prioritize malaria control activities). Thirty-eight villages at least two hours distance away from the nearest selected villages were then identified.

The study population included villagers currently living in the endemic area or its surroundings for at least one month.

### The study design

**Figure 1:**
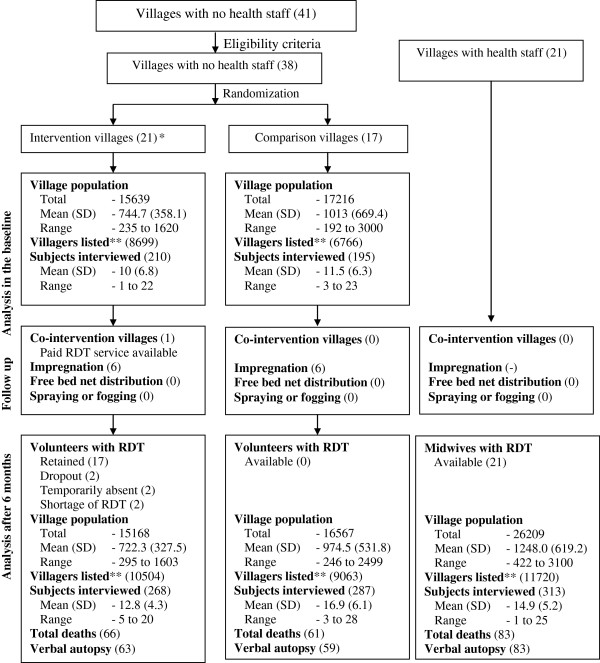
Study flow chart. *Two did not receive training but included as intention-to-treat analysis basis. **Villagers listed in household form to identify eligible subjects for interview.

A cluster randomized controlled trial, with each village as the cluster, was chosen because the volunteer was accessible to all villagers in any given village. Eligible villages (clusters) without existing health staff were then randomized into intervention and comparison groups. The nature of the intervention allowed neither allocation concealment nor blinding. Villages with midwives (MW) in post were used as a second comparison group in the endline survey (see Figure 1). Village characteristics and mortality data were collected and house-to-house survey carried out in the two groups without health staff at baseline and in all three groups at endline. Information from the endline survey was supplemented by an analysis of volunteer logbooks, patient records completed by volunteers, midwives’ logbooks and field-based verbal autopsies.

### The intervention

The intervention consisted of training unpaid village volunteers in the provision of RDT and ACT and supervising them. The VBDC programme supplied volunteers with Paracheck Pf® (Orchid Biomedical Systems, Goa, India), a RDT for the diagnosis of *P. falciparum* malaria with up to 100% sensitivity and a specificity of 75% to 98.3% [3,31,32]. Patients who tested positive were treated with a combination of 20 mg artemether-120 mg lumefantrine (Coartem®). Patients with fever (with or without chills) whose test results were negative were administered chloroquine to suppress *P. vivax* malaria, after excluding local infection. The programme did not provide paracetamol, an antipyretic commonly used and available locally to treat fever.

### Training module and content preparation

A volunteer training module for malaria in Myanmar (see Additional file 1) and the Job Aid illustration on the use of RDT, recommended by the WHO were used [18,33]. The training topics covered communication skills, the nature, symptoms and modes of transmission of malaria, fevers, vulnerable populations, illustrations of RDT, treatment regimens, patient referral, prevention and control measures, thermometer reading, record-keeping and reporting. Referral to basic health staff was recommended for infants and pregnant women, and in the presence of symptoms such as sore throat, difficult urination, ear discharge, cough, loose motion and skin ulcers. The training did not cover the treatment of non-malarial diseases.

Educational flip charts were available in local languages and distributed to volunteers to educate patients. Malaria supervisors also demonstrated the impregnation of mosquito nets. Practice on the use of flip charts and impregnation of mosquito nets was not included in the training.

### Selection of community volunteers

For health volunteers to meet eligibility criteria, they had to reside in the village, be of either gender, 18 years and above, having completed primary school and easily accessible to villagers, willing to work on a volunteer basis, accepted and respected by villagers, and willing to work under the supervision of health staff. In each village, the leader selected a volunteer who was then approved by the health staff.

### Training workshop

The training was held on 19–20 May, 2009 in the Bago Divisional Office. The Bago VBDC team included a malariologist, entomologist, laboratory technician, health education specialist, malaria supervisors; they facilitated the training with practical demonstrations and exercises. Information on volunteers’ background and willingness to serve was collected a day before the training and volunteers were provided with training modules. A self-administered, semi-structured questionnaire was used to evaluate the training.

Volunteers were reimbursed for their travel costs (7.5 to 15 USD) and meals (17 USD) during their stay in Bago. Giveaways included a T- shirt, a cap and a handbag with a malaria volunteer logo.

### Assessment of competence of the volunteers in using RDT and ACT

Following the training, a 16-item WHO RDT training evaluation instrument [33] was used as well as three sets of RDT sample results on a computer screen; this was done until each volunteer performed a RDT proficiently, correctly interpreted its result, and appropriately selected drug treatment and dosage.

### Materials and the tasks to perform

After completing the training, two boxes of Paracheck Pf® RDT (50 packets), AL and chloroquine were provided to all volunteers. Other materials included disposable gloves, a lancet, cotton, spirit, and a timer-watch, thermometer, calendar, patient record forms and a logbook. Monthly calendar cards were issued to record the availability of volunteers and their meetings with health staff. On returning to their villages, volunteers were to inform village leaders, seek their support in endorsing and promoting the programme, display two posters announcing the availability of free RDT, and report to local health staff for future supervision.

### Monitoring and supervision

Midwives in the nearest health facility routinely monitored and supervised volunteers during their immunization visits to villages (monthly). Malaria supervisors occasionally visited volunteers. The first author assessed the regularity of activities through monthly review of calendar cards and reports and regularly visited these townships to collect forms from malaria supervisors but did not supervise their activities.

### Assessments

Data on village information and a listing of household members to screen for fever were collected. Subjects who had reported fever were interviewed by research assistants. Close relatives of the deceased were interviewed using the standard verbal autopsy guidelines [34]. Volunteers’ and midwives’ logbooks were also reviewed.

### Village profile

Background characteristics, village population and malaria control activities were collected from village leaders.

### House-to-house surveys

The baseline survey was conducted in the intervention and comparison villages between 8 March and 6 April, 2009. The endline survey was conducted in all villages visited for the baseline assessment, and in villages with midwives, between 21 November, 2009 and 17 February, 2010. Two teams consistently collected data for both baseline and endline surveys. They consisted of three research assistants from the Department of Medical Research - Lower Myanmar (DMR-LM) for the interviews, and a local health staff for the blood test.

Two pre-tested forms (a household form and an individual form) were used. The household form included village name, township name, a list of household members and their background characteristics such as age, sex, occupation, history of fever during the previous 30 days. The individual form included background characteristics, knowledge of malaria symptoms, number of febrile episodes, date of the episode, place of residence at the time of the episode, treatment type and place received, awareness of the availability and location where free RDT-based treatment was available, history of RDT and the main reason for use or non-use of RDT. The interval between the first symptom (fever) and blood test was assessed for timeliness of RDT use. Data in the endline survey were validated against information from volunteer logbooks.

For each survey, all households were visited and data collected for the household forms. Villagers with high- or middle-school education completed the household forms under the supervision of a research assistant. All household members above the age of six months, living in the village for over 30 days and having contracted a fever within the previous month were eligible for interview. Those with fever for less than 24 hours were excluded, as they could not be assessed for timeliness of RDT. Appointments were made with eligible subjects for a face-to-face interview. When eligible subjects were under the age of 15, the mother or a close household member, were interviewed.

### Ascertainment of mortality for the intervention period

During the baseline survey, village leaders were asked to keep a death register and ascertain the date of death in the endline survey. When reviewed however, the death registers were unavailable or incomplete. Dates of deaths were identified in several ways during the endline survey. First, the list of the deceased after 21 May, 2009 was collected from midwives, village leaders and the informal social group registers. Second, the close relatives of the deceased were requested to recall events on the day of the death of a kin. Third, the dates of death were confirmed with the death notices distributed locally. When a death notice was not available, recall was facilitated by the use of the Myanmar calendar, which includes a festival day every month.

### Verbal autopsy survey

After identifying the deceased during the intervention period, the international standard verbal autopsy guidelines were used to identify causes of death in the post-intervention assessment, using questionnaires appropriate for each age groups [34]. Two doctors and one non-medical assistant interviewed the close relatives of the deceased. The hospital record forms and investigation results of the deceased were also checked with relatives, when available. An interviewer transcribed the information into the verbal autopsy questionnaire. Being responsible for the hospital and public health performance, the township medical officer is responsible for reporting malaria-related deaths. In the verbal autopsies, local health staff and unlicensed practitioners were asked about the symptoms of the deceased such as fever, blood pressure, oedema and tightness of chest.

Two physicians, blinded to the intervention status, identified the causes of death from the verbal autopsy questionnaires. When a discrepancy between their conclusions arose, the final diagnosis was made by a senior doctor from the study area. All were independent from the control programme. Using the standard WHO case-definitions for malaria [35,36], diagnoses were categorized into deaths from probable malaria, non-malaria and fever of unknown origin. Malaria was recorded as a cause of death if complications similar to those observed for malaria had occurred before death. Non-malaria deaths were recorded as diseases other than malaria. Fever of unknown origin was recorded for deaths from fever with no complications mimicking serious malaria, where blood test results were unknown.

### Other reviews

Volunteers’ and midwives’ (18) logbooks and patient record forms were reviewed from 21 May, 2009 until the end of the survey to determine trends in caseload and use of RDT.

### Main outcomes of interest

The primary outcome measures were: (i) whether villagers had received blood test-based treatment for fever, (ii) interval between onset of fever and the blood test, (iii) mortality. The secondary outcome variable was knowledge of malaria mode of transmission.

### Sample size

The proportion of people anticipated to have received treatment within 24 hours after the intervention period was 50% in the comparison arm and 70% in the intervention arm, based on the 2005 Roll Back Malaria target of at least 60% of malaria cases receiving appropriate treatment within 24 hours of the onset of symptoms and the unavailability of information on proportion receiving malaria diagnosis within 24 hours from onset of fever information in Myanmar [35]. The initial screening of household members for fever during the pre-test in two villages and additional qualitative information from local malaria assistants was used to draw an expected mean cluster size of 15, with cluster size ranging from five to 20 and an intra-cluster correlation 0.1. A total of 17 clusters with 257 individuals each were required for the intervention arm and the comparison arm at 5% level of significance and 80% power [37].

### Data analysis

Descriptive statistics including percentages, mean and standard deviations were derived from volunteer background data, logbooks and patient record forms.

The impact of the training was assessed using intention-to-treat analysis method, where two villages in the intervention group were included in the intervention arm although these villages did not receive training of volunteers. “R” software [38] and installed packages were used for the analysis (epicalc [39], survey [40], MASS [41], lme4 [42]). Statistical comparisons were made between two groups (intervention and comparison) and between the two surveys to test the main hypothesis. All three groups (intervention, comparison and MW groups) were included in computations for the modelling.

### Survey analysis for house-to-house survey data

The surveys were analysed using sampling weights to compute estimates and standard errors for outcomes. Sampling weights were calculated using the inverse of the probability of selection, or the estimated total village population divided by the number of villagers screened for fever.

The analysis of knowledge in interviewed subjects who had contracted fever used a scale from zero to eight for eight means of transmission, with a correct answer for each mode of malaria transmission scoring one.

To establish significant differences between the groups (intervention *vs* comparison) and surveys (baseline *vs* endline), the first and second-order Rao-Scott corrections to the Pearson chi-squared test for proportions and two sample *t*-test for continuous variables were used.

### Multivariable analysis

Due to limitations in the survey package in model fitting for hierarchical data, generalized, linear mixed model with random intercepts was employed to predict the binary outcome variable (diagnostic blood test for malaria) with the individual (subjects with febrile illness) as the first level and the cluster (village) as the second level. Akaike’s information criterion (AIC) value was used in the selection of the best-fit model.

### Other analyses

The mortality rates were computed as the ratio of the number of deaths and the mid-survey period population.

### Ethical approval

The Ethical Review Committee, Department of Medical Research (Lower Myanmar) - No. 4/Ethics/2007 and the Ethics Committee, Prince of Songkla University, Thailand - EC 52-054-18-6-2 approved this study.

### Trial registration

Australian New Zealand Clinical Trials Registry ACTRN12610000706077.

## Results

Characteristics of the volunteers and their assessment of the training workshop (see Additional file 2) are not included in the manuscript.

### Characteristics of villages (intervention *vs* comparison *vs* midwives group)

Villages in the intervention arm were smaller in population size than those in the comparison arm and where the midwives resided (see Figure 1) and they were closer to health facilities than villages in the comparison arm (Table 1).

**Table 1:** Baseline and endline characteristics of villages and villagers who had fever within 30 days

	**Baseline**		**Endline**		
	**Intervention**	**Comparison**	**Intervention**	**Comparison**	**Midwives**
**Villages**	21	17	21	17	18
Distance to health facility (in km)					
Mean (SD)	3.5 (2.6)	4.6 (3.5)			
Range	0.2 - 11.3	0.2 - 12.9			
**Villagers interviewed**	(*n* = 210)	(*n* = 195)	(n = 268)	(n = 287)	(n = 313)
	% (SE)	% (SE)	% (SE)	% (SE)	% (SE)
Age group (years)					
Up to 5	6.8 (2.2)	4.9 (1.5)	18.9 (2.7)	8.5 (1.8)	9.7 (1.7)
> 5 to 15	12.6 (2.6)	10.2 (2.3)	16.7 (2.4)	18.9 (2.6)	19.7 (2.5)
>15 to 30	38.5 (4.7)	39.4 (4.7)	28.1 (2.9)	38.2 (3.2)	23.6 (2.6)
>30 to 50	34.1 (4.5)	28.5 (4.4)	21.7 (2.6)	22.0 (2.7)	29.2 (2.8)
> 50	8.0 (2.1)	16.9 (3.2)	14.6 (2.2)	12.4 (2.3)	17.8 (2.4)
Mean (SE)	27.8 (1.2)	30.9 (1.4)	24.7 (1.2)	26.5 (1.2)*	29.0 (1.3)
Sex (Male)	71.7 (3.8)	67.2 (4.4)	57.3 (3.2)*	56.6 (3.3)	61.7 (3.0)
Occupation (over 15)					
Forest worker	24.9 (4.3)	22.0 (4.3)	23.5 (3.2)	26.6 (3.3)	24.4 (3.2)
Farmer	19.7 (4.7)	19.6 (4.6)	28.1 (3.6)	18.2 (2.9)	20.4 (2.8)
Forest and farming	40.5 (5.4)	37.0 (4.9)	28.6 (3.6)	26.3 (3.3)	30.5 (3.5)
Others	14.8 (3.8)	21.4 (4.4)	19.8 (3.1)	28.9 (3.7)	24.6 (3.1)
Education (over 15 years of age)					
Illiterate	6.2 (1.7)	4.2 (1.6)			
Primary	67.3 (5.2)	62.6 (5.3)			
Middle	17.4 (4.3)	19.6 (3.9)			
High and above	9.1 (3.5)	13.5 (4.8)			
Married (over 15 years of age)	59.4 (4.5)	58.2 (4.5)			
Race (Bamar)	70.0 (4.0)	95.1 (1.3)			
Religion (Buddhist)	78.4 (3.8)	97.8 (0.9)			
Being in forest within one year	78.3 (3.7)	73.3 (4.0)			
Has bicycle	29.9 (4.6)	34.4 (4.7)			
Has motor cycle	12.7 (4.1)	9.3 (3.3)			

* Significantly lower than the baseline result.

The trial intervention and comparison groups were similar in the main occupation being forest-related work (14/21 *vs* 10/17), availability of electricity (1/21 *vs* 0/17), availability of schools (20/21 *vs* 16/17), primary level schools (18/21 *vs* 13/17) and water wells as the main source of drinking water (19/21 *vs* 16/17).

### Volunteer dynamics and village co-intervention

The study flow chart is shown in Figure 1. Of 21 intervention villages, two villages did not receive training due to a delay in communication from local health staff. One volunteer dropped out a week after training for personal reasons. Another did so after two months to avoid a personal conflict with a relative receiving temporary, paid RDT from PSI/Myanmar. All 21 villages were included in the intervention group in the analysis. A volunteer who attended the training was identified as a surrogate from an unselected village. He and his village were not included in the analysis but the findings on volunteers’ assessment of the training workshop include his observations (see Additional file 2).

Six villages in each group had benefited from bed net impregnation programmes by PSI/Myanmar. However, no spraying was reported in villages. None of these co-interventions took place in the villages of the midwives.

### Period of intervention (from the day of training to the endline assessment)

The number of days between the start of the project to the endline survey ranged from 183 to 275 days (overall median = 217 and IQR = 201; 237). There was no significant difference in the duration of the intervention in different groups.

### Results from analysis of service logbooks and patients’ record forms of volunteers and midwives

#### Information from the volunteers’ logbooks

All logbooks were available and submitted to the programme monthly. A total of 906 patients was registered by volunteers. Twenty six records were misplaced. Nine volunteers had at least one-month period without any RDT activity. It is not known whether this inactive period was due to his/her absence, RDT shortage or no case of fever.

Logbook data are summarized in Table 2. There were more patients tested per midwife than per volunteer and more positive test rates. Patients in both groups were predominantly male and over three quarters of patients were adults. A quarter of all patients seen by the volunteers and half of those seen by the midwives were patients from other villages. In total, 17 out of a total 71 patients in one volunteer’s register were from a comparison village in the neighbouring township.

**Table 2:** Information from logbooks of volunteers and midwives during the study period*

	**Intervention villages (19)**		**Midwife villages (18)**	
	**Tested**	**Positive/Tested (%)**	**Tested**	**Positive/Tested (%)**
Total registered patients	906		1525	
**Number of patients tested by volunteer**				
Median (IQR)	50 (18-71)		61.5 (35.5-101)	
Range	2-105		18-257	
**All patients analysed**	880	381/879 (43.3)	1525	750/1523 (49.2)
	*n (%)*		*n (%)*	
Sex				
Male	606 (68.9)	278/606 (45.9)	1077 (70.6)	581/1075 (54.0)
Female	273 (31.0)	103/273 (37.7)	448 (29.4)	169/448 (37.7)
Missing	1 (0.1)	-	-	-
Age group (years)				
< 1	3 (0.3)	1/3	7 (0.5)	3/7 (42.9)
1 to 5	44 (5)	26/44 (59.1)	35 (2.3)	14/35 (40.0)
> 5 to 10	75 (8.5)	35/75 (46.7)	105 (6.9)	26/105 (24.8)
> 10 to 15	76 (8.6)	36/76 (47.4)	94 (6.2)	38/94 (40.4)
> 15	674 (76.6)	280/674 (41.5)	1283 (84.1)	669/1283 (52.1)
Unknown	8 (0.9)	3/8	1(0.1)	0
Village where the patient resided				
Same (intervention/midwife)	650 (73.9)	284/650 (43.7)	683(44.8)	345/683 (50.5)
Comparison village	17 (1.9)	8/17 (47.0)	-	-
Other villages not enrolled in study	183 (20.8)	80/183 (43.7)	-	-
Others, not specified	-	-	842(55.2)	405/842 (48.1)
Missing	30 (3.4)	9/30 (30.0)	-	-
Duration of fever before blood test	(n = 737)		(n = 1440)	
Mean (SD)	5.03 (4.81)		3.2 (1.84)	
Median (IQR)	4 (2,6)		3 (2,4)	
Range	1-30		1-21	

* Comparison villages omitted from the analysis due to no patients registered.

Of 381 positive results, eight received inappropriate chloroquine treatment, and seven inappropriate chloroquine treatment was given by a female volunteer. Of 498 negative results, eight were over-treated with AL, and five were given by an unlicensed practitioner who used RDTs from the dropout volunteer when PSI/Myanmar discontinued the RDT supply on a paid basis.

#### Information from patient record forms

Volunteers filled in case record forms for 89.9% (815/906) patients registered in their logbooks albeit leaving blank answers for a number of questions. Case recording data showed that 33.9% (260/768) patients were in the forest at the first episode of fever, 51.8% (342/660) cases self-medicated prior to consulting with volunteers, 1.5% (10/673) presented with poor health conditions and 40 were referred.

When comparing timeliness of diagnosis from volunteer patient record forms with midwives’ logbooks, the number of days between the first day of fever and the day of consultation was significantly longer in intervention villages (Mean 5.03, SD 4.8; Median 4, IQR 2–6) than in villages with midwives (Mean 3.2, SD 1.8; Median 3, IQR 2–4) (*P* value from Mann–Whitney *U* test = 0.00001).

The number of cases tested declined over the six-month period in both volunteer and MW groups but more sharply in the volunteer group. The positive test rate was slightly higher in the MW group. These rates fluctuated over time (Table 3).

**Table 3:** Blood test carried out by volunteers and the midwives during six-month period

	**Intervention villages (19)**		**Midwife villages (18)**	
	**Tested**	**Positive (%)**	**Tested**	**Positive (%)**
Total in six months	795		1382	
Month (from 21 May to 20 November 2009)				
First	279	(47.0)	326	(57.4)
Second	201	(39.8)	309	(47.6)
Third	148	(35.1)	234	(40.6)
Fourth	64	(62.5)	164	(47.6)
Fifth	62	(43.5)	169	(50.9)
Sixth	41	(36.6)	180	(52.8)

### Findings from the surveys

#### Characteristics of respondent villagers who had had fever (intervention *vs* comparison *vs* MW group)

As seen in Table 1, baseline characteristics age, sex, occupation, education, marital status and history of being in the forest in the past year were similar in both groups (intervention *vs* comparison). However subjects in the intervention group were less likely to be Bamar and Buddhist. At endline, the proportion of children was greater in all three groups when compared to the baseline. In all three groups, over half of the patients were solely engaged in forest-related work or also working as farmers.

### Outcomes of the study

Out of 210 deaths identified by village heads and families approached by the research team, three verbal autopsies were unsuccessful in the intervention group and two in the comparison group. Mortality data are summarized in Table 4. Baseline *vs* endline mortality could not be compared because of differences in data collection procedures. Death rates were not different in the two groups in both baseline and endline surveys. Fever-related deaths accounted for approximately one third of all deaths, while one fifth was due to probable malaria. The proportion of deaths from fever and probable malaria were lower in the midwife villages (Table 4).

**Table 4:** Comparison of baseline and endline mortality, fever prevalence and hospital admission for all three groups

	**Baseline**		**Endline**			
	**Intervention**	**Comparison**	**Intervention**	**Comparison**	**Odds ratio (95% CI)**	**Midwives**
**Mortality analysis**						
Total village population	15,639	17,216	15,168	16,567		26,209
Deaths within 30 days from all causes^a^	7	12				
Deaths during survey period (per 100,000 population year, SE)						
All causes			66^c^ (713, SE 88)	61 (606, SE 77)	1.18 (0.82, 1.70)	83 (521, SE 57)
Deaths from fever			20 (216, SE 48)	20 (199, SE 44)	1.09 (0.56, 2.14)	12 (75, SE 22)
Probable malaria^b^			12 (130, SE 37)	12 (119, SE 34)	1.09 (0.45- 2.66)	8 (50, SE 18)
**Period prevalence of fever**						
Villagers listed by survey team	8,699	6,766	10,504	9,063		11,720
	% (SE)	% (SE)	% (SE)	% (SE)		% (SE)
Fever						
Within one month	6.6 (0.39)	7.2 (0.46)	5.0(0.23)	6.1 (0.28)	1.22 (1.06, 1.39)*	4.1(0.19)
Within 3 months			9.4(0.30)	9.8 (0.34)	1.05 (0.95, 1.16)	7.6(0.26)
**Hospital admission from any cause**			0.67(0.08)	0.7 (0.09)	1.03 (0.72, 1.47)	0.8(0.09)

^a^ as reported by village leaders, no verbal autopsy done for baseline survey.

^b^ Based on WHO standard malaria case definitions [35,36].

^c^ Verbal autopsy revealed that all dead cases had never visited the volunteer.

* Significant difference between the intervention and the comparison.

Period prevalence of fever was available for one-month and three-month periods in all baseline and endline surveys. Fever rates in the MW villages were lower than the other two groups in the endline survey.

Hospital admission rates from any causes were similar in the intervention and the control group.

Data on coverage and timeliness of blood testing, knowledge of blood tests and malaria transmission, and health behaviours are summarized in Table 5. At endline, the proportion (SE) of subjects reporting fever in the last 30 days, whose blood was tested for malaria was only 14.9% (2.4%), 5.7% (1.7%) and 21.4% (2.6%) in the intervention, comparison and MW villages, respectively. Blood test coverage in the baseline survey was lower in the intervention village but not significantly so. Although neither group showed an increase in testing rates at endline, the rate in the intervention group was 2.6 times significantly higher. This was around two thirds the rate in the MW group, which was also low (21%).

**Table 5:** Baseline and endline coverage and timeliness of blood test, knowledge and health behaviours

	**Baseline**		**Endline**			
	**Intervention**	**Comparison**	**Intervention**	**Comparison**	*P*value**	**Midwives**
	(*n* = 210)	(*n* = 195)	(n = 268)	(n = 287)		(n = 313)
	% (SE)	% (SE)	% (SE)	% (SE)		% (SE)
**Blood test**						
Blood test coverage	7.2 (2.2)	8.4 (1.9)	14.9 (2.4)	5.7 (1.7)	0.003	21.4 (2.6)
Timeliness in days in Mean (SE) among those tested	4.06 (0.9)	2.86 (0.3)	3.62 (0.35)	4.84 (1.32)	0.37	3.22 (0.43)
**Knowledge**						
Know the place of blood test	39.8 (4.6)	57.0 (4.6)	73.5 (2.8)*	63.2 (3.2)	0.017	80.3 (2.3)
Know the need to check	46.1 (4.6)	52.9 (4.7)	68.1 (3.0)*	61.8 (3.2)*	0.24	76.9 (2.5)
Know free treatment for malaria	12.0 (2.9)	28.8 (4.5)	56.2 (3.2)*	32.0 (3.0)	0.0000	56.8 (3.1)
Knowledge score on transmission (out of maximum possible 8) in mean (SE)	2.40 (0.2)	2.67 (0.1)	2.52 (0.13)	2.40 (0.12)	0.51	2.70 (0.13)
**Health behaviour**						
Has mosquito net	98.2 (0.8)	97.7 (0. 8)	98.5 (0.6)	98.7 (0.6)	0.82	98.8 (0.5)
Has impregnated net	14.6 (3.5)	35.9 (4.8)	35.1 (3.2)*	16.2 (2.2)*	0.0000	29.3 (2.7)
Sleep under bed net	-	-	81.3 (2.4)	71.8 (2.9)	0.01	84.1 (2.3)

* Significant difference from the baseline result.

** Testing difference between the intervention and the comparison group.

**Figure 2:**
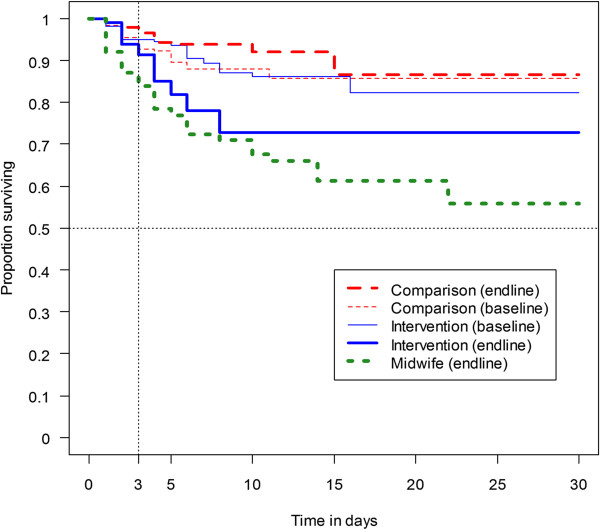
Kaplan-Meier Curve (weighted estimate) for receipt of blood test at baseline and endline surveys.

In all surveys, the mean delay in getting blood test among the tested was around three to four days. Prevalence for receiving a test within 24 hours did not meet the recommended 60% coverage of the WHO. Figure 2 shows a Kaplan-Meier curve for different groups at baseline and endline. The low coverage and the lack of timely use of RDT are reflected in these curves. The post-training curve for the intervention group at the end of survey (thick continuous line) was close to the curve for the MW group. It was distinctly steeper than the baseline pre-training curve of the same group and the two comparison group curves (baseline and endline).

At endline however, those who knew to request a malaria blood test significantly increased from the baseline in both intervention and comparison groups while knowledge about the service location and free malaria treatment increased significantly in the intervention group but not in the comparison group.

Of a total 868 respondents who participated in endline interviews in three groups (intervention, comparison and MW group), 116 (13.4%) reported receiving blood test-based treatment, 89.1% were tested by RDT and 68/116 (58.6%) reported the detection of malaria parasites. Anti-malarial treatment was prescribed to users without a blood test (32 in intervention, 33 in comparison, 20 in MW group) by volunteers in two cases in the intervention group, by local health staff in five cases in the MW group and by unlicensed practitioners in 28 cases for all three groups (11 in intervention, 12 in comparison and five in MW group).

Results of the malaria transmission knowledge assessment with the WHO-modified questionnaire were low throughout the study. All mean scores were below three out of the maximum possible score of eight.

The percentage of villagers with simple mosquito nets was almost 100% and the use of impregnated bed nets was low (<35% at the endline) in all groups. Compared to baseline, the number of villagers who impregnated their bed nets had significantly increased at endline in the intervention group while it significantly decreased in the comparison group. Both ownership of an impregnated bed net and sleeping under a bed net were significantly greater in the intervention than the comparison group.

### Multilevel, multivariable analysis of the endline result

Results of multivariable analysis suggest that villages with a midwife or a volunteer had a greater chance of receiving a blood test than those without either. The odds of receiving RDT in subjects served by the two types of provider were not significantly different. Middle-aged men and those with impregnated bed nets received a malaria blood test more frequently than other groups (Table 6).

**Table 6:** Multilevel modelling predicting receipt of blood test among 868 villagers at the endline survey

**Variables**	**Adjusted OR (95% CI)**
Type of village	
Comparison	1
Midwife	5.40 (2.65 - 11.01)^a^
Intervention	3.19 (1.51 - 6.76) ^a^
Age group (years)	
Up to five	1
5 to 14	1.91 (0.62 - 5.84)
15 to 29	4.98 (1.81 - 13.72)
30 to 49	3.59 (1.29 - 10.01)
50 and above	2.04 (0.66 - 6.28)
Sex	
Female	1
Male	2.07 (1.28 - 3.34)
Ownership of net treated with insecticide	
No	1
Yes	1.80 (1.10 - 2.93)

^a^ Not significantly different from each other.

## Discussion

The intervention resulted in higher RDT coverage in the treatment villages, although an increase was also observed in the comparison village through contamination (receiving a test from the volunteer). The number of patients tested by volunteers declined in contrast to the stable number of patients tested by midwives. In all villages, timeliness of the intervention did not comply with WHO guidelines (over three days after onset of fever). There was no significant difference between groups in overall and probable malaria mortality rates. The low knowledge of patients was not improved by the intervention. However, the increase in the use of bed nets and reduction in the prevalence of fever observed was significant in the intervention group only.

This study was based on cluster randomized controlled trial design that could minimize potential bias and confounders. Blinding used in randomized controlled trials on drug treatment was not possible because the training must be done openly. Exclusion of villages with security problems and those with too small population was unavoidable and it can be a limitation of this study. However, given that this is a relatively small proportion of the population in the study, the limitation should be of serious concern. The results of the randomization process were not perfect as mean distance from home of the villagers to the health facility in the comparison group was around 1 km, far more than that of the intervention group. However, the results of multivariable analysis showed that the outcome was not significantly influenced by the distance and so the distance could not explain the difference between the intervention and the comparison groups.

The higher coverage of RDT after intervention may be explained by the increased availability of RDT, improved knowledge of free treatment and a low contamination (receiving RDT from volunteers) in the comparison group. The lower coverage of villagers in the comparison and intervention groups than in the midwife group may result from a number of factors: midwives had served villagers for longer, non-receipt of training in two intervention villages, unavailability of RDT service from the dropouts and shortage of RDT supply in the intervention group. The findings on the uneven availability of the programme are consistent with findings from similar volunteer interventions in Laos and Zambia [21,27].

While low coverage was observed in this study, higher coverage (40%) found in a previous study may be explained by the fact that half the subjects were from Bago Township where a VBDC office is located, that RDT facilities were available in most villages and hospitals in a few [43]. In other words, remote villages with higher endemicity, especially villages without any health staff are at a greater risk of malaria-related morbidity and mortality. From this study, it is concluded that using volunteers specifically trained on malaria can improve coverage in villages without health staff, although coverage remained low. Innovative interventions should be further explored to improve coverage and knowledge.

Both the coverage of testing and time to blood test were low in all three groups. Such delays can lead to severe malaria [1] with treatment after 48 hours being associated with mortality [2]. Findings related to delays in receiving anti-malarial treatment in this study were similar to those reported in Ethiopia [44] and Yemen [45]. To date, no studies have reported success in increasing the promptness in diagnosis to fewer than 24 hours from onset of fever.

The malaria mortality rates in this study are higher than statistics reported for most Southeast Asian countries. The rates in the intervention and comparison villages were greater than the overall malaria mortality rate (34 per 100,000 population) for Myanmar, which is comparable to the midwife group [46]. The lower malaria mortality rate of Myanmar in WHO reports may be underestimated as a result of incomplete information. A national representative mortality survey in India raised similar concerns related to underestimates of WHO-reported annual malaria deaths [47].

Possible reasons for the absence of difference in overall and probable malaria mortality (from verbal autopsies in the endline survey) may include the relatively small study population, short follow-up, low coverage, insufficiently high endemicity for high mortality or a lack of malaria immunity among villagers. Declines in mortality have been reported in Thailand [19], Ethiopia [23] and Uganda [28] but were not attributed to the use of RDT alone, as the effect of other effective measures, such as impregnated bed nets, could not be ruled out and some studies lacked a comparison group. Results from the verbal autopsies in this study confirmed that no death occurred after consulting a volunteer. In Nigeria, where volunteers were assigned for the treatment of multiple diseases, 13 deaths were reported in 1,028 patients (53% were young children) consulting volunteers for various diseases including malaria [24]. The situation in Myanmar and Nigeria being different, the findings do not indicate that the use of volunteers to treat multiple diseases increases the risk of mortality for malaria-specific diagnosis and treatment. In this study, mortality survey at the baseline and endline employed different techniques. This limitation should be taken into account.

A co-intervention can potentially present a problem for the intervention trial. Using a cluster randomized design, the proportion of villages receiving impregnated bed nets was comparable in both groups, suggesting therefore that the co-intervention was not a likely confounder. While the co-intervention was spread similarly in both study groups, the use of impregnated nets was higher after the intervention, as was the knowledge of facilities for blood test and free malaria treatment. The training programme may have improved the volunteers’ communication skills to participate in impregnation of bed nets. In contrast, the decrease in the ownership of impregnated bed nets in the comparison group may result from the villagers’ knowledge of the six-month lifespan for bed net impregnation to remain active against a longer study period. Despite the presence of volunteers, ownership of impregnated bed nets in the study villages was much lower than that reported in studies from Ethiopia [44], Thailand [48] and other countries in Africa [49].

The number of patients tested by volunteers showed a declining trend. The seasonality of malaria could be a possible cause as malaria normally peaks in June-July and the intervention programme was launched in May. The greater decline in the volunteer group than the MW group may result from a possible unavailability of RDT facility, the absence of volunteers or a shortage of RDT in the volunteer group; an actual reduction in the number of fever cases could not be excluded. Volunteers’ absence may be seasonal, work- or study-related or the result of decreasing motivation over time for being unpaid. Shortages of RDT were due to delays in delivering supplies to volunteers, resulting from frequent staff transfers at the township level, limited numbers of malaria staff at the township level and the lack of budget for supervisory visits. The shortage of drugs supplied to community health workers in Zambia was found to be associated with a loss of reputation and subsequently low performance [27]. While midwives checked patients from different villages, cross-village testing by volunteers was limited. This may be due to insufficient information sharing among villages regarding the availability of the volunteer programme.

More male patients with fever were reported as were higher RDT positive rates than in females. These differences may be the result of work in the forest leading to increased exposure to malaria or other factors, such as a vulnerability to particular strains of the malaria parasite [50]. In this study, knowledge of malaria was similar for both genders. Differences in access to RDT may be due to the perceived level of difference in reasoning among the genders for malaria screening or other gender-related diseases. This needs further study.

Compared to volunteers, midwives tested fewer younger people even though test results show a high positive rate. This may be explained by the integrated role of midwives in overseeing maternal and child health related issues. Children and young people are known to be more vulnerable to severe consequences from malaria and need more attention [51,52].

Other studies in Nepal [53], Kenya [54], Peninsular Malaysia [55] and Myanmar [56] have reported low knowledge of malaria. In this study, the low or irregular use of educational materials such as flip charts by midwives and volunteers and uneven distribution of pamphlets in the intervention group may partly explain this finding.

Paracheck-Pf*®* RDT has been recommended in Myanmar for its high sensitivity and high prevalence of *P. falciparum* in the country. The findings in this study show high rates of *P. falciparum* malaria (43% of cases tested by volunteers and 49% by midwives). The limitation of Paracheck-Pf*®* RDT in detecting *P. vivax* malaria does not answer the question of the magnitude of non-fatal malaria. Presumptive treatment for a negative result with chloroquine does not eradicate *P. vivax* malaria. A 14-day primaquine regimen was not prescribed to avoid possible, serious adverse reactions. This could lead to a predominance of *P. vivax* malaria in this area in the future. It is hoped that with technological advances RDT will also become available for *P. vivax* malaria allowing them to be treated simultaneously.

## Conclusions

This study demonstrates that the coverage of RDT can be improved by implementing a volunteer programme. However, the use of RDT was still low and that tests were not performed in a timely manner and that the number of patients tested declined over time. The use of impregnated nets and the knowledge of malaria were found to be low in all groups. The supervision component of the programme needs to be strengthened for quality control and sustainability of the programme.

## Abbreviations

(MW): Midwives; (RDT): Rapid diagnostic test; (ACT): Artemisinin-based combination therapy; (AL): Artemether-lumefantrine; (VBDC): Vector Borne Disease Control team; (WHO): World Health Organization; (PSI/Myanmar): Population Services International/Myanmar; (SD): Standard deviation; (SE): Standard error; (IQR): Inter-quartile range; (OR): Odds ratio; (95% CI): 95% confidence interval.

## Competing interests

The authors declare that they have no competing interests.

## Authors’ contributions

O, TM, SS, TW made contribution to conception and design of the study. VC made substantial contribution to revise the design. O, TM, SS carried out data collection and data cleaning. O and VC were involved in data analysis, interpretation and manuscript preparation. All authors read and approved the final manuscript. O is the guarantor of the paper.

## Supplementary Material

Additional file 1 **Training module on malaria volunteers.** The training module on malaria volunteers is a translated version of the one available in Myanmar language. Click here for file

Additional file 2 Background information about volunteers and their assessment on training workshop. Click here for file
