# Supplementary material for: Effects of malaria volunteer training on coverage and timeliness of diagnosis: a cluster randomized controlled trial in Myanmar
Source: Malar J. 2012 Sep 4;11:309. doi: 10.1186/1475-2875-11-309 (PMC3488026; doi:10.1186/1475-2875-11-309)
Supplement: Additional file 1 — Training module on malaria volunteers. The training module on malaria volunteers is a translated version of the one available in Myanmar language. [file 1475-2875-11-309-S1.doc]

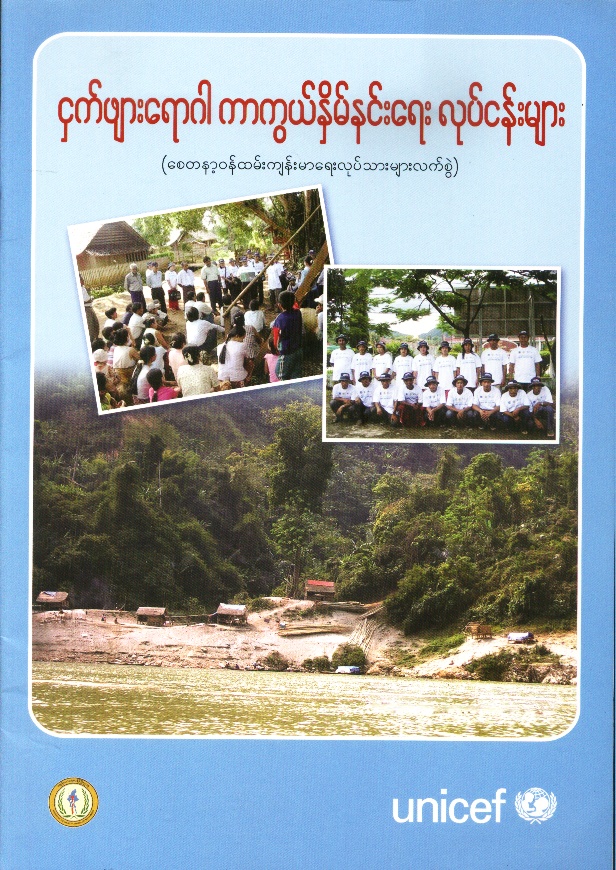


**Malaria Prevention and Control Activities**

**(Handbook for Volunteer Health Workers)**

**Malaria Prevention and Control Activities**

**(Handbook for Volunteer Health Workers)**

**Handbook on malaria prevention and control for**

**social organizations/ volunteer health workers**

**Content**

**Malaria Prevention and Control Activities**

**(Handbook for Volunteer Health Workers)**

| **No.** | **Topic** | **Page** |
| --- | --- | --- |

[Introduction 1](#__RefHeading___Toc322196374)

[1. The Malaria Crisis 3](#__RefHeading___Toc322196375)

[Learning objectives 3](#__RefHeading___Toc322196376)

[What is malaria? 3](#__RefHeading___Toc322196377)

[Causes of malaria 3](#__RefHeading___Toc322196378)

[Incorrect beliefs about malaria 4](#__RefHeading___Toc322196379)

[Malaria parasite 5](#__RefHeading___Toc322196380)

[Mosquito which carries malaria parasite 6](#__RefHeading___Toc322196381)

[Breeding habitat of anopheles mosquito 6](#__RefHeading___Toc322196382)

[Mosquito breeding areas 6](#__RefHeading___Toc322196383)

[Anopheles mosquito breeding sites 6](#__RefHeading___Toc322196384)

[Blood-feeding behaviour 7](#__RefHeading___Toc322196385)

[Mosquito life span 7](#__RefHeading___Toc322196386)

[Vulnerable populations 8](#__RefHeading___Toc322196387)

[Symptoms of malaria 9](#__RefHeading___Toc322196388)

[Severe malaria symptoms 9](#__RefHeading___Toc322196389)

[Causes of fever which can be mistaken with malaria fever 10](#__RefHeading___Toc322196390)

[2. Malaria Prevention and Control 13](#__RefHeading___Toc322196391)

[Basic information on malaria prevention and control measures 13](#__RefHeading___Toc322196392)

[3. Health Education and Community Participation 19](#__RefHeading___Toc322196393)

[1. Advocacy to village leader after training 22](#__RefHeading___Toc322196394)

[2. Advocacy to villagers after training 23](#__RefHeading___Toc322196395)

[3. Health education and discussion on early diagnosis and effective treatment by interpersonal communication 24](#__RefHeading___Toc322196396)

[4. Prevention of mosquito bite 27](#__RefHeading___Toc322196397)

[5. The use of flip chart for health education 30](#__RefHeading___Toc322196398)

[4. Community Organizing 31](#__RefHeading___Toc322196399)

[5. Reporting of an Outbreak 33](#__RefHeading___Toc322196400)

[Blood test for malaria with the Rapid Diagnostic Test (RDT) 33](#__RefHeading___Toc322196401)

[Guidance for early diagnosis and effective treatment of malaria 36](#__RefHeading___Toc322196402)

[Coartem® dosage 37](#__RefHeading___Toc322196403)

# Introduction

Malaria is a health problem as well as a socioeconomic problem. People with in low socioeconomic conditions are affected more. Worldwide, there are 300 to 500 million cases of malaria every year with approximately one to two million deaths. In Myanmar, there have been approximately 700,000 reported cases of malaria with 2,700 deaths on average each year for the past five years. Nineteen hundred new cases of malaria and 8 deaths are reported daily, with majority of the victims being men of economically productive working age. Malaria, therefore, not only affects the patient and the patient’s family but also has negative impacts on the economic growth of a nation.

The World Health Organization (WHO) adopted the Roll Back Malaria (RBM) policy to assess the magnitude of the problem and to reduce the occurrence of malaria. It set a target to reduce the incidence of morbidity and mortality caused by malaria in the year 2000 by 50% by the year 2010.

In the RBM strategy, people faced with malaria are required to participate individually or as a group in malaria prevention and control activities. Participation of social organizations and volunteer health workers is crucial to the success of this strategy and to the improvement of the health of the community. Promoting **the role of social organizations and volunteer health workers** in malaria prevention and control activities is therefore important not only for the benefit of the community; it will also have an impact on the socioeconomic development of the country. It is therefore essential that health workers have knowledge of malaria prevention and control strategies, capability to carry out malaria prevention and control activities and willingness to in volunteering their service for the benefit of the community. The success of community-wide programs requires active community participation and support in public health services. The burden of malaria can be reduced through participation of all.

In RBM, **key responsibilities** of social organizations and volunteer health workers are:

- To provide health education to community and to organize people to participate in public health activities
- To impregnate bed nets with net treatment and to promote proper utilization of bed nets
- To help with early diagnosis and treatment of malaria and to refer patients to hospitals and clinics
- To report immediately to the health centre when there is unusually high occurrence of malaria or febrile patients in the community.

This handbook has been developed to provide guidance to social organizations and volunteer health workers for effective participation in malaria prevention and control activities.

Chapter 1 provides detailed information about the disease

Chapter 2 provides simple and easy steps to control and prevent malaria

Chapter 3 provides detailed information on provision of health education to the community and organizing community for malaria prevention and control activities. The important activities are listed in annexes.

# The Malaria Crisis

**Malaria in your village**

### Learning objectives

Participants will understand

- What is malaria?
- Causes of malaria
- Spread of infection
- Malaria and vulnerable populations
- Symptoms of malaria

### What is malaria?

Malaria

- is an infectious disease spread by mosquitoes and caused by the parasite *Plasmodium*, which infects blood cells.
- affects approximately 1900 people and causes eight deaths every day in Myanmar.
- treatment is costly in the event of serious disease condition.
- is also a socioeconomic problem.
- causes a loss in family income due to lost days at work for the patient and for those who take care of the ill patient*.*
- has negative impact on household income and overall economy.

### Causes of malaria

Malaria is caused by the bite of a mosquito that is infected with the malaria parasite. It is transmitted when the mosquito bites a person after biting a person infected with the malaria parasite. Mosquitoes that cause malaria bite at night. The malaria parasite is so tiny that it can only be seen under the microscope. Malaria parasites can live in the human host and anopheles mosquitoes. Parasites invade red blood cells in human and causes malaria symptoms.


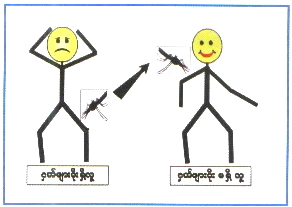


He has

malaria parasite

He does not have malaria parasite

### Incorrect beliefs about malaria

Malaria cannot be caused by

- drinking or bathing spring water
- eating bananas, papaya or bamboo shoot
- spirit or ghost or mythical belief


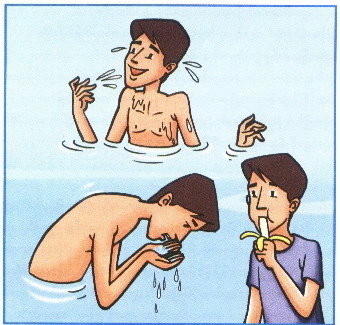


Malaria parasite

Malaria parasite is a plasmodium parasite. There are four types of plasmodium parasites that can cause malaria. They are

1. *Plasmodium falciparum*
2. *Plasmodium vivax*
3. *Plasmodium malariae*
4. *Plasmodium ovale*


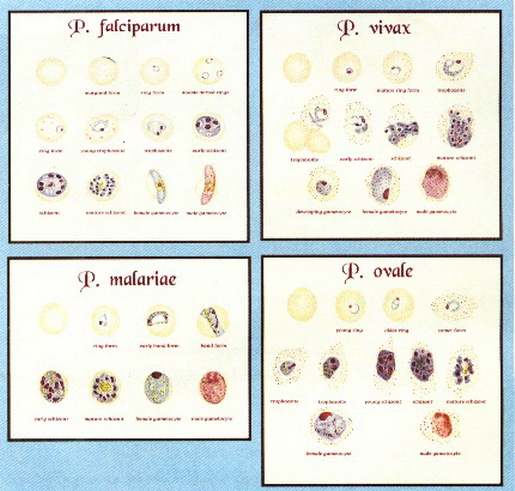


### Mosquito which carries malaria parasite

- Malaria is transmitted by the bite of a mosquito.
- The mosquito which carries the malaria parasite is known as anopheles mosquito.
- Female anopheles mosquitoes transmit malaria by biting a person after having bitten a person infected with malaria parasite, thus making malaria an infectious disease
- Anopheles mosquito usually bites from dusk to dawn.


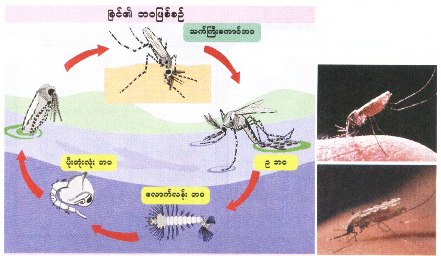


**Life cycle of mosquito**

**Adult mosquito**

**egg**

**larva stage**

**pupa stage**

### Breeding habitat of anopheles mosquito

### Mosquito breeding areas

- coastal regions, forest areas
- forest fringes and plains with agricultural irrigation.

### Anopheles mosquito breeding sites

Anopheles mosquitoes breed in

- places where there is standing water such as bamboo hollow, tree hollow, stone cavities, cow footprints in mud, hand pumped ground water, shallow well and lake.
- paddy field, standing water in agricultural farms, water leaks from dams and water gutters
- open sun-lit pools with slow running water
- wells located in the fruit gardens in slum areas and villages in Mon State, Kayin State and Tanintharyi Region
- wells located in agriculture farms in Twin Taung area in Butalin Township, Sagaing Division
- ponds and lakes in the coastal zone.

### Blood-feeding behaviour

- Female anopheles mosquitoes feed on human or animal blood for successful fertilization of its egg.
- They usually feed on human blood once in two days.
- Male anopheles mosquitoes do not feed on human blood.
- Only female anopheles mosquitoes feed on human blood and transmit malaria in humans

### Mosquito life span

- The life span of a female anopheles mosquito is about one month.

### Vulnerable populations

Malaria can affect males and females of all ages, of all ethnicities. However, people who are most vulnerable to malaria include:

- workers who work in forestry-related jobs (wood or bamboo cutters, charcoal makers) mine workers (gold miners, petroleum mining workers)
- workers in newly established business worksites in malaria endemic areas (those working in roads construction, construction of bridges and dams, electricity generating industries, ministry of mines, agricultural farming and new village settlements)
- pregnant mothers and children in malaria endemic areas
- people who live in malaria endemic areas and those who do not sleep under mosquito net at night time

**The most vulnerable people for malaria**


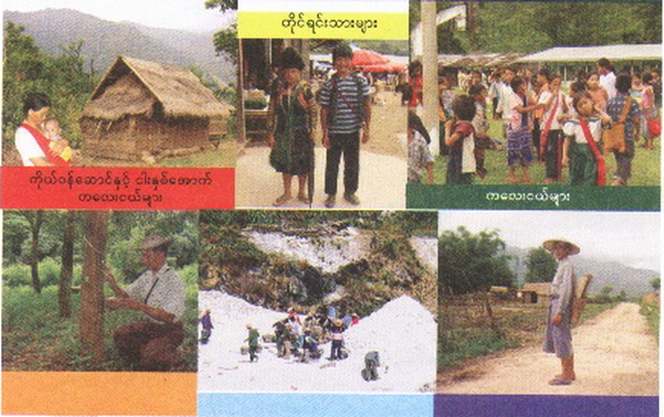


**Ethnic people**

**Children**

**Pregnant mothers and**

**Children under the age of 5**

### Symptoms of malaria

- Malaria can present as a fever with chills and sweating. Fever can occur in alternate days or in frequent episodes.
- In the beginning stage of malaria, fever can occur daily.
- Other symptoms are
  - headache, loss of appetite, dizziness, vomiting, muscle and joint pain, abdominal pain, pallor due to anaemia, spleen enlargement and pain, and frequent cough and loose motion.
  - If left untreated or treated inadequately, the disease can be severe and can lead to death.


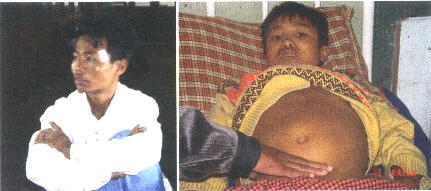


### Severe malaria symptoms

- Unable to stand up without someone’s help, unable to sit, unable to walk
- Severe vomiting which results in failure to take treatment orally
- Extremely high fever
- Delirium
- Severe drowsiness
- Mental confusion
- Convulsion, unconsciousness
- Aggressive behavior
- Bleeding, passing black coloured urine
- Severe anaemia, eye pallor
- Cold and clammy extremities, signs of shock
- Breathlessness
- Scanty urine

Note: Other diseases can also present symptoms like malaria.

### Causes of fever which can be mistaken with malaria fever

#### Influenza

- It occurs during the influenza season. Symptoms are fever, feeling cold, headache, body ache, runny nose, cough and sore throat.

#### Typhoid

- Fever is continuous and not intermittent.
- Pulse rate is slow although there is fever. Tongue is coated.

#### Hepatitis or Jaundice

- There can be chills at the onset of fever. Other symptoms are loss of appetite, nausea, vomiting, dark yellow coloured urine, pain in right hypochondrium region and yellow eyes.

#### Meningococcal meningitis

- It occurs in summer. Symptoms are neck stiffness, sudden rise of fever that can result in death within a few hours, severe muscle pain, severe headache and skin rash (red and purple spots).

#### Elephantiasis

- It is common in lymphatic filariasis endemic areas such as lower Sagaing Division, Magwe Division and Yakhine State.
- Signs and symptoms are fever with chills, hardening and thickening of skin, tightness of chest, edema in limbs, pain and swelling of scrotal sac in males, swelling of scrotum and passing of rice-water coloured urine.

#### Urinary tract infection

- Symptoms are fever with chills, difficulty in urination, scanty amount of urine, increased frequency of urination, painful urination and dark coloured urine.

#### Puerperal fever

- Symptoms are fever with chills and rigor after delivery or within 42 days of abortion.

Therefore, blood examination is necessary to differentiate between malaria and other causes of fever.


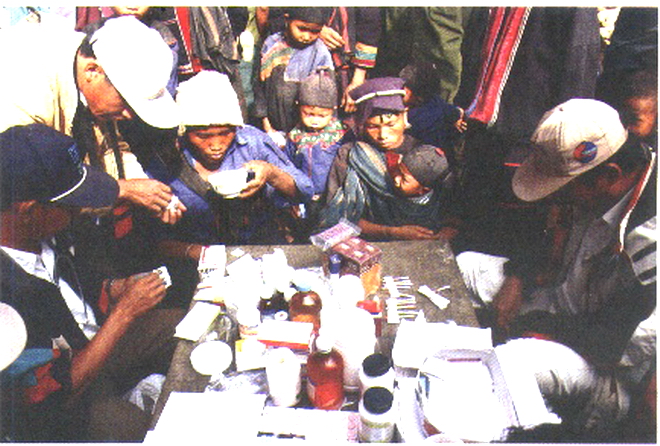


#### Summary on malaria

| - Malaria is not only a disease but also a socio-economic problem. It can be fatal. It makes poor people poorer. - Malaria is infectious but it is not transmitted through eating bananas, papaya or bamboo shoot, drinking or bathing spring water or by spirits or ghosts. It is transmitted by the bite of an infected female Anopheles mosquito. - It is caused by a malaria parasite. - Anopheles mosquitoes which carry malaria parasites usually bite from dusk to dawn. - Malaria can occur in coastal regions, forest areas, forest fringes and near the forest. - Malaria can occur in males and females of all ages and ethnicities. - Malaria occurs more commonly among those who do not sleep under mosquito net or those who work in the farm or forest at night time - Symptoms of malaria are frequent febrile episodes, feeling cold and chills and sweating. Fever can occur in alternate days. Fever may occur daily in the beginning stage.   Other symptoms are headache, loss of appetite, dizziness, vomiting, muscles and joints pain, abdominal pain, pallor due to anaemia, spleen enlargement, often cough and loose motion.   - Other diseases can also present symptoms like malaria. All fevers with chills are not due to malaria. - Malaria can be diagnosed by a blood test. |
| --- |

# 2. Malaria Prevention and Control

### Basic information on malaria prevention and control measures

**Learning objectives**

After reading this chapter, volunteer health workers should become familiar with the following topics:

- malaria prevention and control measures
- the importance of the daily use of mosquito net for sleeping at night
- the importance of impregnation of bed net
- the importance of early diagnosis and proper treatment for malaria,
- importance of collaboration with the community by organizing people through health education.

Malaria is caused by *Plasmodium* parasite. It is transmitted by the bite of an infected female anopheles mosquito. Malaria can cause many problems.

Therefore, it is necessary to understand the following malaria prevention and control measures to solve problems related to malaria.

1. Personal protection from mosquito bite
2. Prevention of mosquito breeding in the environment
3. Control of mosquitoes
4. Prompt and proper treatment of malaria patients


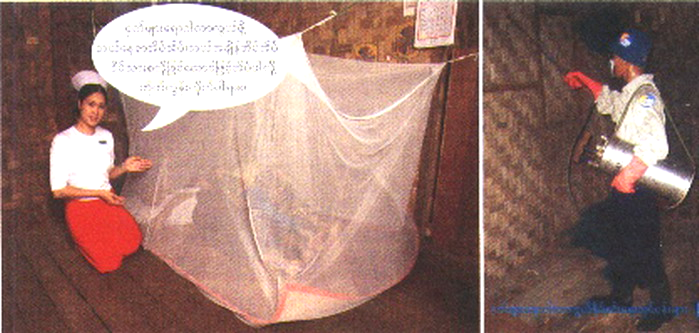


**At any time or at any place, I suggest you to sleep under the family net to prevent malaria.**

1. Personal protection from mosquito bite

| No. | Measures | Advantage | Disadvantage | Remark |
| --- | --- | --- | --- | --- |
|  | Sleeping under simple bed net | Can prevent mosquito bite; Possible long term use | Slightly costly | Good |
|  | Sleeping under impregnated bed net | Can prevent malaria better than the simple net; Possible long term use | Needs regular impregnation; Slightly costly | Good |
|  | Wearing thick clothes in long sleeves for prevention of mosquito bite | Method is easy. | Difficult to comply in the hot season | Fair |
|  | Use of mosquito repellent | Can prevent mosquito bite | Moderately costly; Effective for about four hours only. | Fair |
|  | Smoke; Burning wood or leaves etc. | Can get rid of mosquito to some extent | Can cause respiratory tract infections; Can affect fetus in pregnant woman | Should not be used |
|  | Mosquito coil | Can get rid of mosquito to some extent | Can cause respiratory tract infections; Can affect fetus in pregnant woman | Should not be used |

See annex on how to do impregnation of bed net

1. Prevention of mosquito breeding in the environment

- Elimination of vegetation (grasses, rushes, reeds) and deposits along banks of shallow creeks, wells and lakes or stagnant water to prevent mosquito breeding
- Actions to improve the flow of water in water creeks and dam tributaries.
- Draining water or land filling of unused ponds which can be potential places for mosquito breeding.
- The use of abate larvicide in anopheles breeding places like ponds and wells. It is a kind of chemical to kill insects and it can kill larva. Because of its high cost and potential environmental pollution, it should be used by trained people when necessary.

1. Control of mosquitoes

- Insecticide spray into the walls can be used to control mosquitoes in houses
- The insecticide kills the mosquitoes landing on the walls sprayed with insecticide.
- Spraying is effective for 3 to 6 months depending upon the type of insecticide. Because of the high cost and environmental pollution, it should be applied by trained people when necessary.
- The community health office staff should perform this activity systematically.
- Volunteer health workers need to work together with the community health workers in organizing people for active community participation.

1. Prompt and proper treatment of malaria patients

- Malaria parasites live in human host and anopheles mosquito.
- Prompt and proper use of recommended treatment of malaria can cure the disease and prevent transmission.
- It is the best to receive proper treatment within 24 hours of onset of fever. The interval between onset of fever and the treatment should not be more than 72 hours.
- Receiving prompt treatment is the only way to prevent severe malaria, cerebral malaria and death. It is important that patients take the full dose and full course of medications for successful treatment.
- As a health care worker, you are responsible for providing complete instructions regarding treatment regimen.

#### Summary

| Basic information on malaria prevention and control measures  - Malaria is caused by malaria parasite and transmitted through the bite of anopheles mosquito. In particular, anopheles mosquito usually bites at night time. - The systematic use of bed net while sleeping at night prevents mosquito bites and malaria. - Impregnated bed nets are not harmful including to children and babies. - Carry out anopheles mosquito control activities such as spraying long lasting insecticides in houses, sanitation of streams, ponds and wells, and the use of larvivorous fish. Malaria prevention and control team should decide on the place and method of spraying. - Prompt and proper use of the recommended treatment of malaria not only cures the disease but also prevents transmission of malaria. |
| --- |

# 3. Health Education and Community Participation

**Malaria prevention and control activities requiring community participation**

1. Sleeping under the mosquito net at all times and all places
2. Impregnation of mosquito net at least once a year
3. Seeking proper treatment for a household member with a fever within 24 hours from skilled health staff or volunteer health workers
4. Elimination of mosquito breeding places in the residential environment as community based group activities

Volunteer health workers will provide information and education to individuals or groups to enhance community participation.

**Health education activities on malaria control**

1. Educate the community about the factors responsible for occurrence and transmission, of malaria and related health problems

- Use pamphlets distributed by Vector Borne Disease Control Team (Central), Department of Health
- Volunteer health workers must read chapter 2 of this handbook and make sure that they themselves understand issues related to prevention and treatment of malaria before talking to the community about these topics.

1. Through discussions about prevention, control and treatment activities with individuals and groups, you make sure that the community is able to undertake these activities on their own.

Read Chapter 3 of this handbook and make sure that you understand the proper procedures before talking to the community members.

1. Inform febrile patients that they can come to you for a blood test and treatment within 24 hours of fever and malaria diagnosis and that you can provide effective drugs for treating malaria.

- Read Chapter 2 of this handbook to make sure you understand correct protocol.

1. Organize and encourage the community to participate in malaria prevention and control activities and to comply with diagnosis and treatment.

**Health education** can be provided to individuals and to members of a group with the use of illustrations, pamphlets, posters and flip charts. The method of impregnation of bed net can be demonstrated.

**Community Health Education and Community Organizing**

#### Information and methods

| No. | Information to be given to the community | Target group | Method | Instrument to be used |
| --- | --- | --- | --- | --- |
|  | Inform the local leaders about the malaria training workshop that you attended. | Village leader | Advocacy | Reference book – chapter (3) |
|  | Inform the villagers about the malaria training workshop that you attended and how you can help them. | Villagers | Advocacy | Reference book – chapter (3) |
|  | Inform villagers that they should seek diagnosis and treatment from you within 24 hours of fever when they get ill. | Villagers | Advertisement | Reference book – chapter (1) RDT and ACT, Flip Chart |
|  | Inform villagers about the use of personal protective measures to prevent malaria | Villagers | Discussion, Health talk | Reference book – chapter (2) Pamphlet, Poster & Flip Chart |
|  | Encourage villagers to sleep under mosquito net at all times; encourage them to impregnate their mosquito nets at least once a year | Villagers, forest workers | Discussion, Health talk,  Demonstrate impregnation of bed net | Reference book – chapter (2) Bed net treatment and practical demonstration |
|  | Encourage community participation in conducting malaria prevention and control activities | Village leader and villagers | Advocacy through leadership of the village leader | Reference book – chapter (2)  Poster & Banner |

### 1. Advocacy to village leader after training

When you (volunteer health workers from social organizations) arrive back to your native villages after completion of the training, meet with village leaders and inform them about your training and the activities that can prevent malaria outbreak in your village. (Read the following paragraph below as an example and discuss about it)

#### The information for advocacy

1. I have attended “**Training workshop on malaria prevention and control for volunteers”** in .........town. As soon as I returned, I am coming to see you (the chairman of the village authority).
2. The training workshop in town lasted for three days and it was conducted by … (Name)… organization. The training workshop provided information about malaria in detail and I have learned how to treat and prevent malaria. I also learned how to do a blood test to check for malaria, how to give antimalarials and how to do impregnation of bed nets.
3. At the training, I received materials to test blood for diagnosis of malaria and some antimalarial drugs for treatment of patients. I am now able to test villagers with fever for malaria and provide them with drugs for treatment.
4. I would also like to explain to the villagers what I have explained to you about the disease. Therefore, I’d like to request you to help organize the villagers for an educational meeting.
5. Thank you for your time and your attention.

After you have given this information, answer the questions asked by the village leader. Keep in mind the date mentioned by the village leader to meet with the villagers. Prepare activities and handouts to explain prevention and treatment of malaria procedures to the villagers.

### 2. Advocacy to villagers after training

After you (volunteer health worker from social organizations) explained the intended activities to the village leader on return to your village, you have to organize an advocacy meeting with the villagers on the date and time as arranged by the village leader. (Read the following paragraph below as an example and discuss it).

#### The information for advocacy

1. I have attended “**Training workshop on malaria prevention and control for volunteers”** in .........town. As soon as I returned from the training workshop to the village, I met the chairman of the village authority. Now, as arranged by the chairman, I am getting a chance to share information with you about what I learned from the training.
2. The training workshop in ………town lasted for three days and it was conducted by the … (Name)... organization. There were volunteer health workers from other villages there as well (If this is true, you should include it in this dialog. If the villagers know that other villages are also doing the same thing, it may increase their desire to learn and follow instructions. They can even confirm from other villages) The training workshop provided volunteer health care workers like me information about malaria in detail and I learned a lot about prevention and treatment of malaria. I learned how to do blood tests for malaria, how to give antimalarials and how to do impregnation of bed net.
3. Materials to test blood for diagnosis of malaria and some antimalarial drugs were given to me after the training. With these things, I can test people with a fever for malaria and give drugs for treatment. Therefore, any person with fever should come to see me within 24 hours. I will do a blood test to check for malaria and give treatment with modern drugs. These drugs are effective. If the treatment is taken early and effectively, it will cure the disease quickly. Then, cerebral malaria and severe malaria will not occur and you can return to work immediately.
4. Treatment for malaria alone is not complete. It is necessary to take precautions so that you do not suffer from malaria again. There are several personal protective measures you can take. Of these, the best is to sleep under a mosquito net at all times. Sleeping under an impregnated bed net can prevent malaria more effectively than a simple bed net. There will be training provided on these activities soon. Till that time, I request you all to sleep under a bed net and contact me in case of a fever. (After you have given this information, answer any questions asked by the villagers. Provide the villagers information about your work place and how they can contact you. For example, your residence)

### 3. Health education and discussion on early diagnosis and effective treatment by interpersonal communication

Interpersonal communication is an effective means to enhance early diagnosis and appropriate treatment. A role play can be performed during the session with the villagers to inform them about malaria, and steps they can take to protect themselves. One participant can act as a villager and another as a volunteer health worker.

**Mr. Aung Aung** – volunteer health worker

**Mr. Khin Shwe** - villager

Mr. Khin Shwe and Aung Aung meet at the tea shop located in the centre of the village and they have a conversation. Other villagers in the tea shop join them and listen to their conversation. Their conversation is described below.

**Shwe** – Hello Aung Aung! I heard from villagers that you attended training workshop in town and that you learned how to treat malaria. Can you tell me about it?

**Aung** – Yes, Uncle Shwe. What a coincidence! I’m trying to find someone to explain. In the training workshop, I learned about the symptoms of malaria, how to check for malaria, how to treat malaria and what antimalarial drugs can be used to treat malaria.

**Shwe** – Oh! Just learning about malaria? I have heard about it too. Fever with chills means malaria. You attended a training to learn about that. I could have told you about malaria.

**Aung** – Uncle Shwe, what you said is not wrong. Malaria is a kind of fever that can present with fever and chills, hot body and sweating etc. Fever occurs frequently or on alternate days. During the initial days, fever may occur daily and the symptoms may be similar to other diseases. The symptoms are headache, loss of appetite, dizziness, vomiting, muscle and joint pain, abdominal pain, anaemia and pallor, spleen enlargement and pain, and occasionally cough, loose motion and so on.

**Shwe** – Wow! A variety of symptoms! Hmm.., many people died from malaria in the past because of just taking home remedies because of unavailability of effective drugs.

**Aung** – Uncle, actually, fever with chills can happen in other diseases in addition to malaria. Therefore, it is the best to take treatment after one has had a blood test to diagnose malaria. If the blood test and treatment are taken within 24 hours of fever, it will prevent cerebral malaria and severe malaria …and a patient can return to work very quickly. When malaria is either untreated or treated ineffectively, then the disease can be serious and as you have seen before, it can often be fatal.

**Shwe** – I agree about the blood test. Is the blood test available with you now? How much will it cost?

**Aung** – Yes, Uncle. It is available with me. The blood test is free. You don’t need to spend a penny.

**Shwe** – If so, what about the cost of drugs? We are lazy and do not take drugs and don’t like to pay for expensive drugs either. We are used to buying medicines we can afford. Will we be able to afford these new drugs?

**Aung** – Uncle, after the training, the drugs were given to me for free. So the treatment is free. You don’t need to spend a penny. You just have to follow the instruction and take the full course of medicines for three days. Only then, the disease will be cured. If someone stops taking the medicine before the full course, the disease will not be cured. There can be relapse and drug resistance, and often it can lead to the death.

**Shwe –** If so, can you treat all malaria patients? When the disease is serious, they do need to go to the hospital, don’t they?

**Aung** - Uncle, I will not treat all malaria patients. Some will need hospital treatment, especially when they have the following symptoms: -

- they are unable to stand, sit or walk without someone’s assistance
- they have severe vomiting and therefore cannot take oral drugs
- very high fever
- delirium
- severe drowsiness
- mental confusion
- convulsion, unconsciousness
- aggressive behaviour
- lack of interest in their surrounding environment
- bleeding, black coloured urine
- severe anaemia and eye pallor
- cold clammy extremities and shock
- breathlessness and
- scanty urine output

I will refer these patients to get treatment in health centers.

**Shwe** – Oh yes! Now, I understand. I will share this very useful information with everyone I know. I will tell them (villagers) to see you within 24 hours of fever for a blood test and full course of treatment when they get fever… and… to go to the hospital when necessary.

**Aung** – Thank you Uncle. If you get fever, you are welcome any time.

**Shwe –** Oh! I want to be in good health. I hope not to come to you..ha..ha..ha..

### 4. Prevention of mosquito bite

#### (Group Discussion)

Volunteer health workers have to give health education on measures to prevent malaria as described in chapter 3 either individually or in a group setting.

1. Encourage people to sleep under simple bed net or impregnated bed net
2. Give the following instructions so that they are able to use bed net properly

The size of the holes in the mosquito net must be small to prevent penetration of mosquitoes. The mosquito net must not have a tear. Carefully secure the corners and the bottom portions of the net under the mat or the mattress. The mosquito net must be sufficiently spacious so that the body does not touch the side of the net. Use a blanket or pillow or clothes between the person sleeping inside the net and the sides of the net.

1. Tell villagers to cover up their body by wearing long sleeve shirts, trousers etc. to prevent mosquito bites while going out at night.
2. Advise villagers to apply topical mosquito repellent on face for temporary protection

Note. The use of mosquito coil and smoke can reduce the abundance of mosquitoes; however, they should not be used because of their adverse effects on respiratory tract and fetus in pregnant women.

**The following short story can be used for a role play among participants to encourage forest workers to use bed net and impregnated net.**

**Mr. Aung Aung** – volunteer health worker

**Mr. San Myint** – **forest worker**

**Ms. Htet Htet** – Mr. San Myint’s sister

**Mrs. Aye Bone** – Mr. San Myint’s mother

The story is about Mr. San Myint, a forest worker who got fever and took blood test from Mr. Aung Aung, a volunteer health worker. The blood test result indicated malaria caused by *falciparum*. Aung Aung treated Mr. Myint with Coartem® antimalarial drug for three days and the disease was cured. Aung Aung wanted to know about the health condition of Mr. San Myint. The following conversation took place while he went to see Mr. San Myint during his stroll in the village.

**Mrs. Aye Bone** – Hello, Aung Aung. Please come and take a seat. My son is now feeling much better. He says he will get into the forest in the next four or five days. So, I am trying to persuade him not to go. I think he should not go into the forest because he is still so frail.

**Aung Aung** – Yes, Mr. San Myint. You should rest for some more days.

**Mr. San Myint** –Malaria is not a strange disease to me because I get it frequently. I suffer from malaria about five times a year.

**Ms. Htet Htet** – That’s why he lost all the money he could earn. He barely survived.

**Mrs. Aye Bone** - I told him not to drink spring water and not to take a bath in spring water too. He didn’t follow what I said. He likes banana and papaya very much and so he gets fever.

**Aung Aung** – Aunty, malaria is not because of drinking or bathing in spring water. The mosquitoes near the forest stream carry malaria parasites. The mosquito bite causes malaria. Malaria is not caused by eating bananas and papaya. When people are bit by mosquitoes, they get infected by malaria parasites, and eating bananas and papayas makes the parasites grow stronger and so the patients get a fever.

**Mr.San Myint** – What you said might be true. In the forest, our tent is located near the stream. At night, we slept there and we were bitten by several mosquitoes.

**Aung Aung** – Brother, when you went into the forest, did you take the mosquito net with you? Did you sleep under the mosquito net?

**Ms. Htet Htet** – My brother has never taken the mosquito net.

**Mrs. Aye Bone** – Mosquito net is costly and I can’t afford to buy one.

**Mr. San Myint** – In the forest, it is not easy to hang the net.

**Aung Aung** – What you all have said may be true for your own reasons… but, if you take into consideration about what I am going to say, it may not be so difficult for you to have a net of your own.

**Ms. Htet Htet** – If you say so, can you please tell us?

**Mr. Aung Aung** – Brother, you said that you got the fever about five times a year. How much was the medical expense each time?

**Mrs. Aye Bone** – The expense ranges from 5000 kyats to 10,000 kyats each time.

**Aung Aung** – Let us say it costs you 8000 kyats each time you get a fever. So, it will cost you 40,000 kyats a year. When you take into consideration of your lost income for not being able to work, and the loss of income of family members for taking care of you, the patient, the expense will be at least 100, 000 kyats each year.

**Mrs. Aye Bone** – Yes, we never calculated the cost like that. Now, we know from your calculation that it is very costly to get malaria.

**Aung Aung** – That’s why I am telling you to invest in a mosquito net. The cost of a mosquito net is around 5000 kyats and it can be used for up to three years. If you calculate, the expense will be just five kyats a day. If you use the net at home as well as in the forest, febrile illness will occur less frequently, maybe one or two times instead of five times a year. You will be able to save money for the times you are free from fever, right?

**Ms. Htet Htet** – If so, Mom…. we should buy a single net for my brother.

**Mr. San Myint** – By the way, how can we do impregnation of bed net? Explain a little to me.

**Mrs. Aye Bone** – Oh! If we use the bed net, isn’t that enough? Is it necessary to do impregnation?

**Aung Aung**  - Your simple bed net can be impregnated with insecticide. When a simple net is used, mosquitoes and other insects may fly near the net and the nuisance may disturb your sleep. The simple net cannot prevent mosquitoes that enter through the holes in the net and bite you. If there’s a tear in the net, mosquitoes can enter the bed net. When it is impregnated, mosquitoes do not come near to the net and you do not get bit by mosquitoes.

**Mr. San Myint** – If so, how much it will cost to impregnate bed net?

**Aung Aung** – A tablet of net treatment costs about 700 kyats. The impregnation is effective for about six months. The expense will be three kyats per day.

**Ms. Htet Htet** – If so,… if a single bed net is purchased and it is used after impregnation, it will cost about eight kyats a day. Eight kyats a day is less than the expense you use every day for smoking, betel nut chewing and drinking toddy palm juice. Smoking, betel nut chewing and drinking palm liquor can affect health. Mosquito net is good for health.

**Mr. San Myint** – What a naughty girl you are! You are saying this as if you are my teacher. If they ask for volunteer health workers in future, I will send you to attend the training workshop. At present, you should buy a mosquito net and net treatment for me before you attend the training. After that, you ask Aung Aung to help with impregnating the net, okay?

**Ms. Htet Htet** – Yes, my elder brother. Mom, please let me have some money soon.

**Aung Aung**  - Htet Htet, when you have mosquito net and treatment tablet, bring them to me. I will do impregnation for you. Aunty, bye! Mr. San Myint, bye for now!

**Mrs. Aye Bone and Mr. San Myint** – Okay! Come back and visit when you are on the way.

### The use of flip chart for health education

In order to promote the use of bed net and blood test based treatment among forest workers, ask individual participants to practice the use of flip chart that was distributed by the World Health Organization (WHO).

# Community Organizing

Health education plays an important role in bringing about change in perception of disease, behaviour and practice observed by the community in seeking treatment for malaria, self protection and participation in malaria prevention and control activities. Malaria prevention and control activities can be performed by an individual or through community-wide activities.

Health education enables community organizing by bringing people together through self-motivation to participate in malaria prevention and control activities.

#### Who is the target population for community organizing?

The whole community especially those living in malaria endemic areas.

#### How to perform community organizing?

Use health education pamphlets and posters; seek assistance of village leaders, local authorities, religious leaders and teachers to bring people together. Organize villager participation through village leaders, religious leaders and respectful community people.

#### Community wide activities for eradication of malaria

- Eliminate mosquito breeding places
- Trim bushes and get rid of standing water near houses to eliminate breeding grounds for mosquitoes
- Build cowsheds between residential homes and mosquito breeding places to reduce human contact with mosquitoes.
- Involve community participation to drain puddles and fill open trenches to eliminate mosquito breeding grounds
- Eliminate debris from lakes, wells and creeks
- Establish a larvivorous fish hatchery to introduce fish in wells and lakes as predators of anopheles mosquitoes.
- Support activities of village people on impregnation of bed net
- Assist malaria inspectors to inspect the proper use of impregnated bed net among villagers.

# Reporting of an Outbreak

When there are an unusually high number of febrile patients or deaths, social organizations and volunteer health workers must inform the nearest health centre immediately either by post or in person. The volunteer health worker must provide villagers proper education on malaria-related topics so villagers can recognize and report outbreaks in villages immediately.

### Blood test for malaria with the Rapid Diagnostic Test (RDT)

#### Step by step protocol

1. Provide the following information to the patient about the RDT

- The blood test device can only detect *falciparum* malaria parasite, which can cause (severe) cerebral malaria, and it will not detect other types of malaria. (Explain that there are four types of malaria parasites.)
- If the patient does not have *falciparum* malaria parasite, fever may be due to other types of malaria parasites or other diseases.
- If the *falciparum* malaria parasite is detected, it is necessary to follow instructions properly in taking oral antimalarial tablets.

1. The use of RDT and the step by step protocol

- Always wear gloves during the blood test to avoid contamination.
- Open the RDT test packet. (DONOT use an already opened test packet.)
- Write down the patient’s name or registration number and the time of examination.


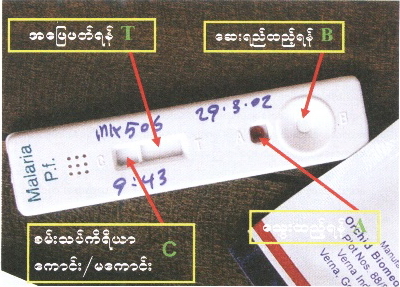


**To read result T**

**For buffer solution B**

**To put blood A**

**To check whether the test is damaged C**

- Use a disposable lancet to collect blood (Use a new lancet for each patient)
- Choose the third or the fourth finger of the patient’s left hand to draw blood.
- Use an alcohol swab to clean the surface of the finger before pricking blood.
- Prick the blood from the side surface near the tip of finger.
- Prick the blood by the rolling movement of your wrist while holding the lancet with index finger, middle finger and the thumb.
- Try to obtain a small required amount of blood in one needle prick


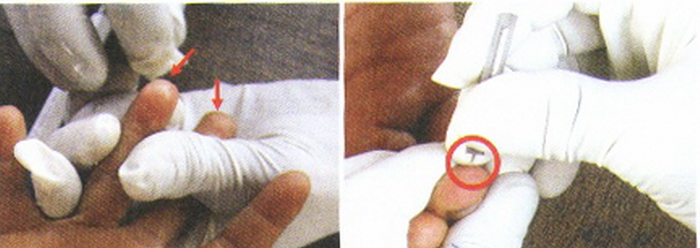


- Use the straw to collect the drop of blood. Then, put the drop of blood into the hole in the cassette for blood.
- The amount of blood must be optimum (The amount of blood must be neither scanty nor abundant)
- When you use dipstick instead of the cassette type device, touch the tip of the straw with blood on the dipstick where the arrow is pointing. Don’t place the tip of straw with blood at the tip of the dipstick because the blood from the tip cannot reach the place where the arrow is pointing.


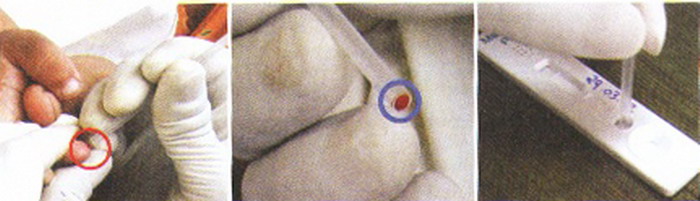


- Put six drops of buffer solution into the round well. (Wait for a while after the first three drops. Then, put three more drops)
- Hold the bottle of solution in a vertical upside down position and drop the solution keeping the tip of bottle at one inch distance from the cassette.


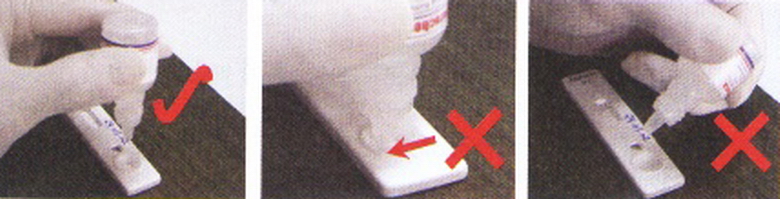


1. Reading the test result

- If the Test Line does not appear, wait for 15 minutes. Read the result under good light. Record the test result. The test line result after 20 minutes is not valid.
- If the Control Line appears only, you can say that *falciparum* malaria parasite is negative. However, fever may be due to non *falciparum* malaria or other diseases. (It is necessary to review symptoms carefully.)
- If both the Control Line and the Test Line appear, the patient has *falciparum* malaria parasite.
- It is necessary to give the recommended antimalarials together with proper instruction.
- If the Control Line does not appear, the test is invalid. It is necessary to use a new one to do the test again.


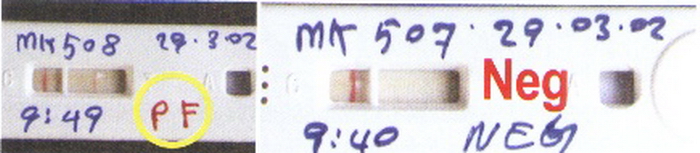


1. Explain the patient after the blood test

- If the result is positive, tell the patient that the result indicates the presence of severe malaria parasite. Before you give antimalarials, ask the patient on the recent use of antimalarials (Mefloquine must not be taken twice within one month). Explain that the disease will be cured when the full course of treatment is taken systematically. Explain that it is important to take the full course of treatment although fever subsides.
- Explain that the patient may suffer from dizziness from taking antimalarials and therefore to avoid working with machines or activities related to the use of machines (for example, vehicles, engines), and to avoid climbing to high places.
- If the result is negative, explain to the patient that the result indicates the absence of severe malaria parasite and it is not necessary to take high potency antimalarials. Explain that it is necessary to take chloroquine if symptoms indicate malaria.

### Guidance for early diagnosis and effective treatment of malaria

Check the following symptoms in those with suspected malaria

Severe vomiting and failure to take treatment orally

Inability to stand up without someone’s help, unable to sit, unable to walk.

Extremely high fever (40°C and above)

Severe drowsiness

Delirium, Mental confusion, Convulsion, Unconsciousness

Bleeding, Passage of black coloured urine

Scanty urine, No urine output

Eye pallor, Jaundice

Severe pallor

Cold and clammy extremities, Signs of shock

Breathlessness

Black coloured stool

Refer infants and pregnant mothers with fever to the nearest health centre, hospital.

The presence of any of the symptoms above indicates a severe febrile condition.

Immediately refer the patient to the nearest health centre, hospital.

If patient cannot reach a hospital within 24 hours and the patient can take oral treatment, give Coartem with proper instruction.

Counsel the patient.

Appearance of two lines in the test, in an indicator of the presence of *P.falciparum* malaria parasite

Give Coartem with proper instruction

Counsel the patient.

If fever does not subside within 72 hours after taking treatment, there can be another disease in addition to malaria.

Refer the patient to the nearest health centre.

Counsel the patient.

The presence of any of these symptoms indicates febrile condition other than malaria.

Refer patient to basic health staff.

Counsel the patient.

Appearance of one line is an indicator of the absence of *P.falciparum* malaria parasite

Fever likely due to non *P.falciparum* malaria parasite or another disease.

Check symptoms of other diseases

Sore throat, swallowing is painful

Frequency and difficulty in urination

Ear discharge

Tightness of chest, Cough

Skin red coloured spots

Loose motion

Skin ulcers, Boils

If none of these symptoms,

it may be malaria.

Give chloroquine in recommended dose.

Counsel the patient.

The absence of all symptoms mentioned above indicates uncomplicated fever.

- Perform blood test with RDT

### Coartem® dosage

#### (Use only in *P.falciparum* parasite positive cases)


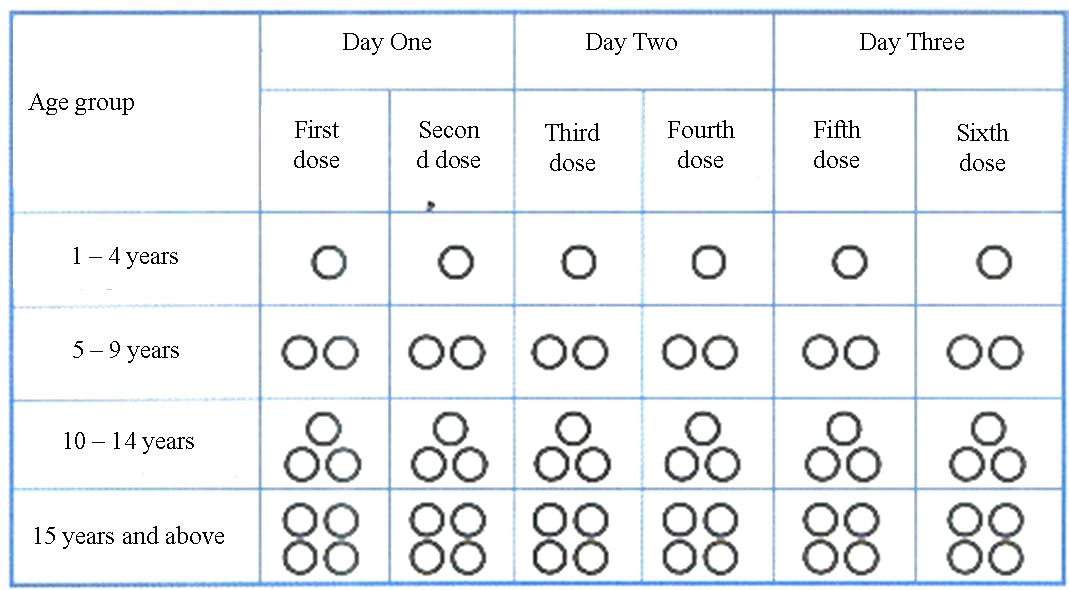


Note. Coartem® must not be given to pregnant women and to children under one year old. It can be toxic. Refer *P.falciparum* positive pregnant mothers and children under one year old to the nearest health centre.

#### Chloroquine dosage (For suspected malaria cases with negative blood test result for *P.falciparum* parasite)


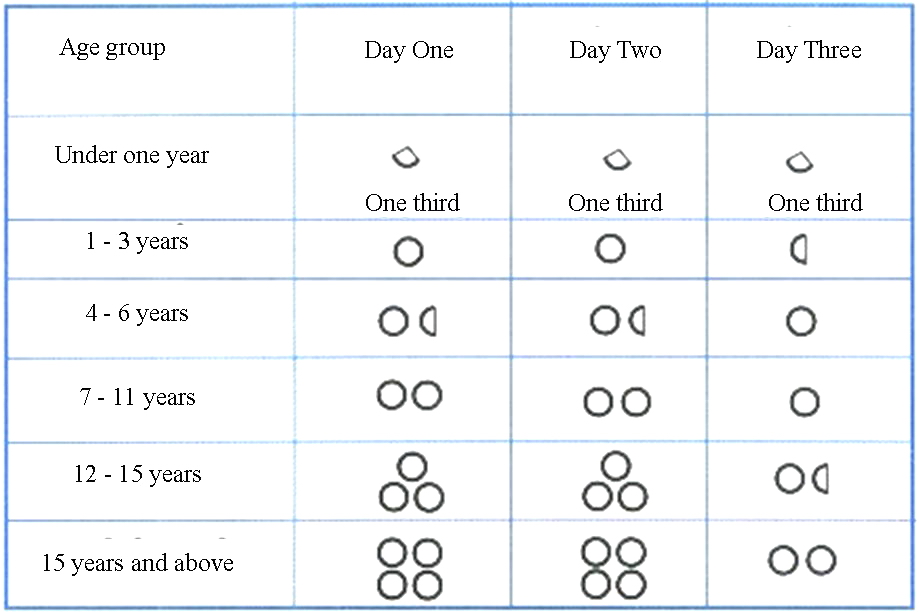


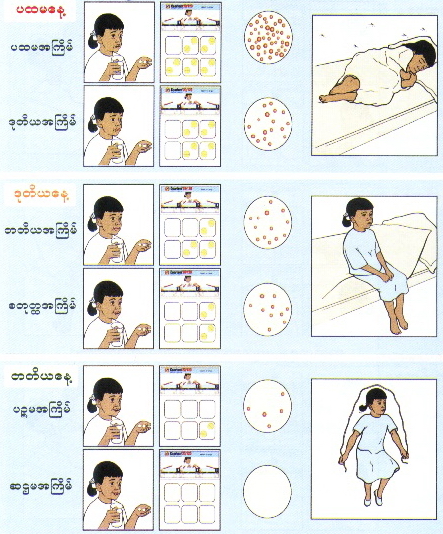


**Follow the instruction to take the full course of Coartem®.**

**The disease will certainly be cured.**

**Day One**

**Day Two**

**Day Three**

**First dose**

**Second dose**

**Third dose**

**Fourth dose**

**Fifth dose**

**Sixth dose**

**Take medicine for full six doses within three days. Although fever subsides, it is necessary to take the full course. Take the whole strip of medicine as in the instruction. Only then will your malaria disease be certainly cured.**


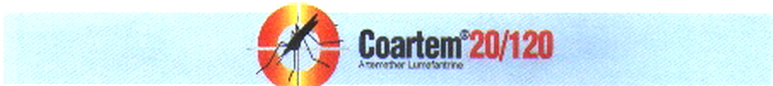


.


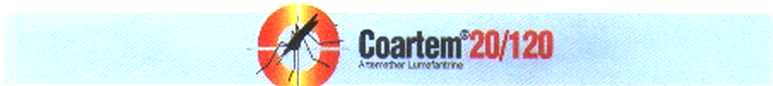

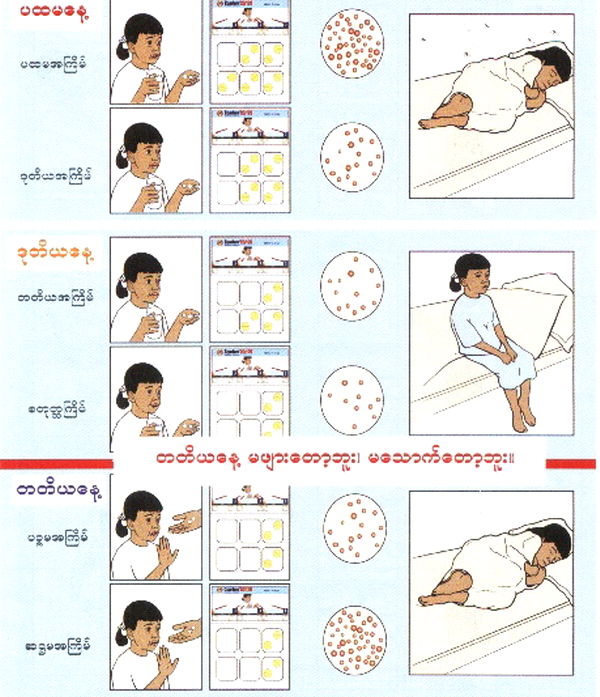


**Day One**

**Day Two**

**Day Three**

**First dose**

**Second dose**

**Third dose**

**Forth dose**

**Fifth dose**

**Sixth dose**

**On Day three, I have no fever. I will not take it.**

.


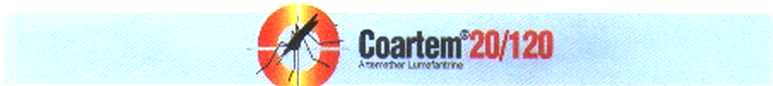

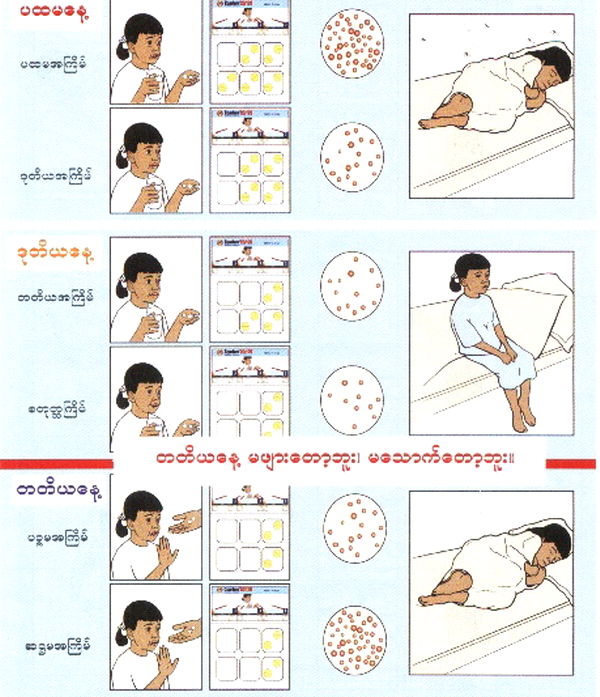


**Day One**

**Day Two**

**Day Three**

**First dose**

**Second dose**

**Third dose**

**Fourth dose**

**Fifth dose**

**Sixth dose**

**On Day three, I have no fever. I will not take it.**

**Follow the instructions to take the full course of Coartem®.**

**The disease will certainly be cured.**

**Take medicine for full six doses within three days. Although fever subsides, it is necessary to take the full course. Take the whole strip of medicine as in the instruction. Only then will you be completely cured of malaria.**

| 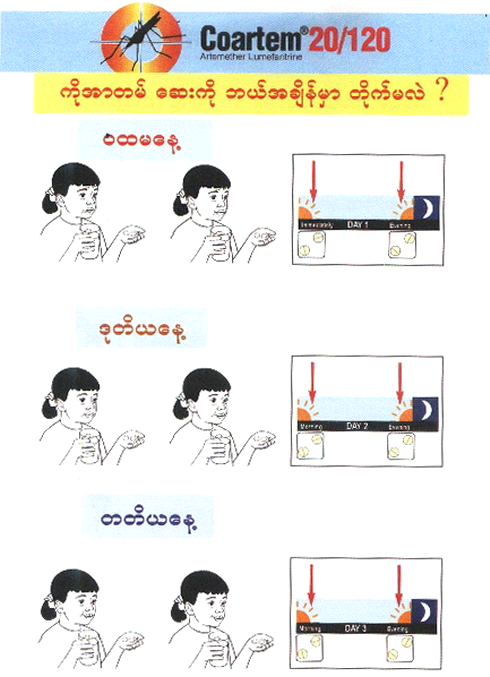 **Day One**  **Day Two**  **Day Three**  **What time**  **Coartem**  **is to be taken?** 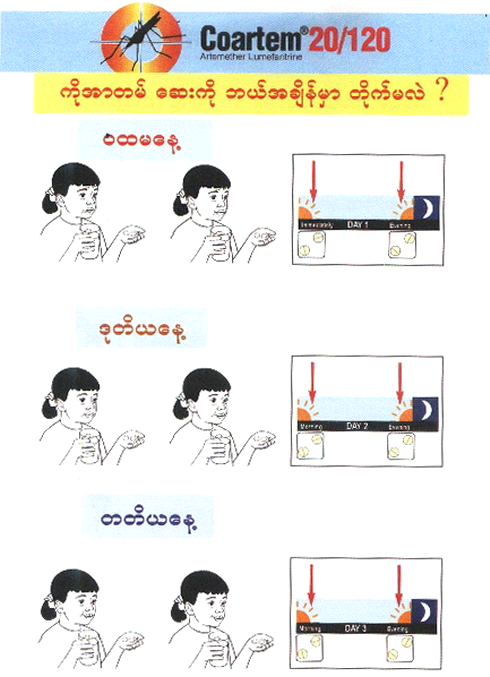 **Day One**  **Day Two**  **Day Three**  **What time**  **Coartem**  **is to be taken?**  **Day One**  **Day Two**  **Day Three**  **What time**  **Coartem®**  **should be taken?**  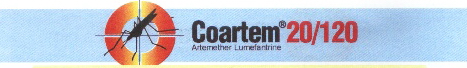  **How to take Coartem®?** | |
| --- | --- |
| **For children, crush the tablet into a powder and mix it with water or milk before giving it to the child.**  **Continue to breastfeed while taking Coartem®.**  **Take Coartem® tablet with juice or milk. If juice or milk is unavailable, take it with water.**  **Malaria may cause loss of appetite. Try to eat as much as possible before taking the tablet. This drug is very effective against malaria.** | 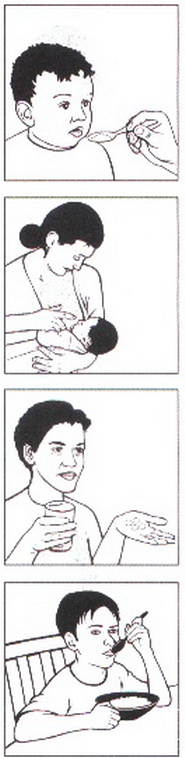 |
