# Supplementary material for: Effects of malaria volunteer training on coverage and timeliness of diagnosis: a cluster randomized controlled trial in Myanmar
Source: Malar J. 2012 Sep 4;11:309. doi: 10.1186/1475-2875-11-309 (PMC3488026; doi:10.1186/1475-2875-11-309)
Supplement: Additional file 2 — Background information about volunteers and their assessment on training workshop. [file 1475-2875-11-309-S2.doc]

### Background information about volunteers and their assessment on training workshop

One volunteer was a surrogate from a village not selected in the study. The background information of 19 volunteers in the intervention villages is shown in the table below.

Of the 20 anonymous participants, 17 perceived the training materials to be sufficient and three perceived them to be moderate. Six volunteers thought that the duration of the training workshop was sufficient, while 14 thought it was moderate. Seventeen volunteers reported that they could understand the training content. Out of the 16 responses to the question as to how to improve the training, the most common (6/16) response was to extend the duration of the training to four or five days.

## Volunteers’ background characteristics, experience, and attitudes towards malaria and the volunteer system

| **Characteristics** | Male (n=10) | Female (n=9) | Total (n=19) |
| --- | --- | --- | --- |
| Age (years) |  |  |  |
| Mean (SD) | 33.6 (8.7) | 21.9 (3.8) | 28.1 (9.0) |
| Range | 18-45 | 18-28 | 18-45 |
| Race |  |  |  |
| Bamar | 7 | 5 | 12 |
| Kayin/Shan/Chin | 3 | 4 | 7 |
| Religion |  |  |  |
| Buddhist | 7 | 7 | 14 |
| Christian/others | 3 | 2 | 5 |
| Education |  |  |  |
| Primary | 3 | 0 | 3 |
| Middle | 4 | 2 | 6 |
| High | 3 | 4 | 7 |
| Graduate | 0 | 3 | 3 |
| Married | 6 | 0 | 6 |
| Occupation |  |  |  |
| Farmer | 9 | 3 | 12 |
| Seller/teacher | 1 | 2 | 3 |
| Dependent | 0 | 4 | 4 |
| **Experience in health and village activities** |  |  |  |
| Non-malaria health training received | 1 | 3 | 4 |
| Malaria training for taking blood slide received | 1 | 0 | 1 |
| Ever treated a patient with antimalarials | 1 | 1 | 2 |
| Responsible in village activities (school, health, social) | 8 | 6 | 14 |
| Supporting local health staff in village health activities | 1 | 4 | 5 |
| **Attitudes towards malaria and volunteer** |  |  |  |
| Malaria is a major health problem in your village |  |  |  |
| Strongly agree | 6 | 7 | 13 |
| Agree | 4 | 1 | 5 |
| Undecided | 0 | 1 | 1 |
| A volunteer should be trained for malaria in your village |  |  |  |
| Strongly agree | 3 | 6 | 9 |
| Agree | 7 | 3 | 10 |
| You agree to attend the training workshop for malaria |  |  |  |
| Strongly agree | 9 | 7 | 16 |
| Agree | 1 | 1 | 2 |
| Undecided | 0 | 1 | 1 |
| People in your village accept you to be a malaria volunteer |  |  |  |
| Strongly agree | 6 | 7 | 13 |
| Agree | 4 | 1 | 5 |
| Undecided | 0 | 1 | 1 |
